# Open-source SX1280 2.4 GHz FLRC transceiver: Overcoming sub-GHz LoRa bottlenecks for high-rate, low-latency continuous telemetry

**DOI:** 10.1016/j.ohx.2026.e00848

**Published:** 2026-10-12

**Authors:** Kent Jojan Carlos Choquepuma, Karla Erika Apaza Rodriguez, Franco Alessandro Arenas Mamani, Alexander Hilario-Tacuri

**Affiliations:** Universidad Nacional de San Agustín de Arequipa (UNSA), Arequipa, Peru

**Keywords:** FLRC, SX1280, ESP32-S3, Long-range telemetry, Open-source hardware, Wireless data acquisition

## Abstract

Transmitting continuous scientific telemetry over remote distances is constrained by sub-GHz LoRa bandwidth limits and regional duty cycles, which cannot sustain 115 200 bps streams. To overcome this, we present OpenFLRC v1.0, an open-source 2.4 GHz FLRC transceiver platform for dedicated point-to-point links. The carrier board contribution is the system-level engineering required to operate the commercial E28 module as a reproducible continuous-duty scientific telemetry instrument: an open carrier board with a 5-capacitor low-ESR decoupling ladder suppressing 550 mA transients, an unbroken bottom ground plane for thermal dissipation without an active heatsink, and dedicated physical RF-switch routing; paired with a FreeRTOS dual-core firmware pipeline isolating 18 MHz SPI DMA transactions to Core 1 while leaving Core 0 largely available for user sensor acquisition. Controlled laboratory benchmarking against 433 MHz and 915 MHz LoRa validates sensitivity, spectral purity (second-harmonic suppression of 38.5–41.8 dBc), and emulates multi-kilometre links via calibrated attenuation. While sub-GHz links collapse under continuous throughput, OpenFLRC sustains 884.3 kbps (PDR≥99.9%) at a 1 ms packet interval under laboratory link budgets. All files are released under CC BY 4.0.

## Graphical abstract

**Figure:**
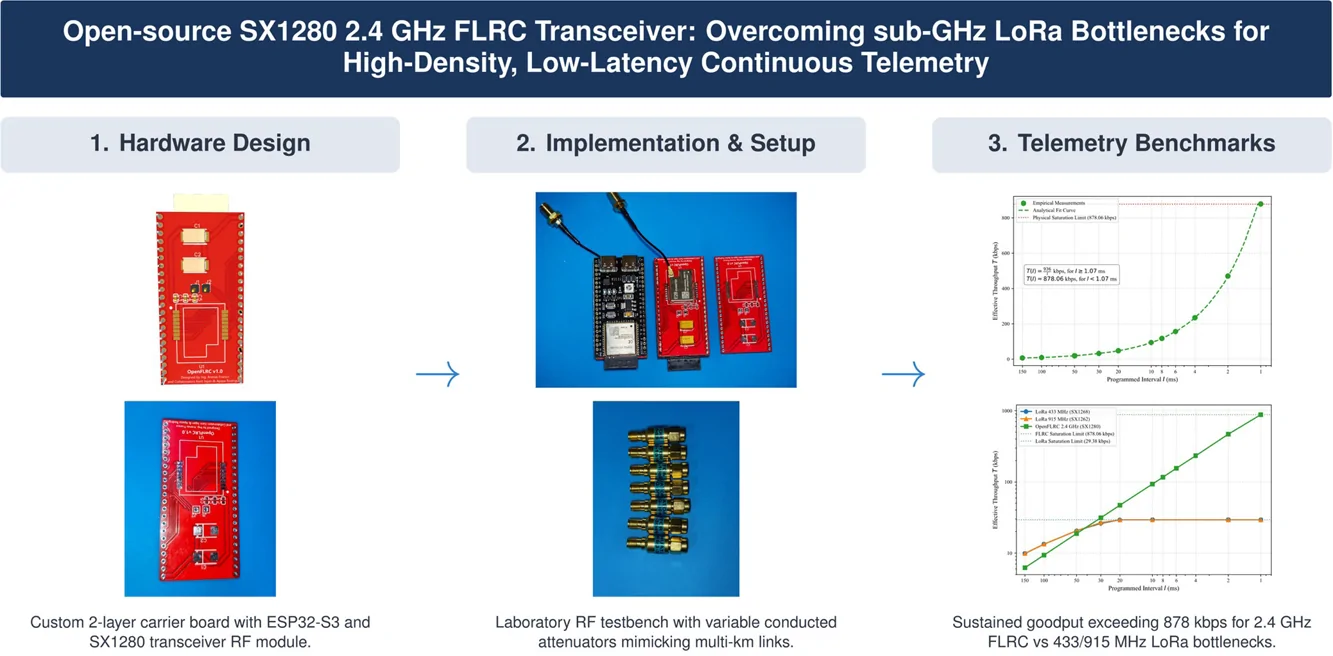

## Specifications table

**Table:** 

Hardware name	OpenFLRC v1.0 – Open-Source 2.4 GHz FLRC Transceiver Node

Subject area	Engineering and material science

Hardware type	Electrical engineering and computer science

Closest commercial analog	Ebyte E22-900T30D/E22-400T30D (Semtech SX1262, sub-GHz LoRa, UART interface). No commercial open-hardware analog combining ESP32-S3 + SX1280 FLRC exists for continuous high-throughput stream telemetry.

Open source license	Creative Commons Attribution 4.0 International (CC BY 4.0) for all design, firmware, and dataset files.

Cost of hardware	$32.70 USD per transceiver node

Source file repository	https://doi.org/10.5281/zenodo.21159006

OSHWA certification UID	PE000005

## 1 Hardware in context

Continuous wireless telemetry of high-resolution scientific data over distances of several kilometres is a persistent challenge in remote field instrumentation. Volcano observatories, seismic networks, and geophysical monitoring stations deployed in areas without cellular or wired infrastructure require reliable, high-throughput radio links that can sustain the uninterrupted data rate of continuous, high-resolution sensors [1]. A triaxial seismometer sampling at 100–200 samples/s (SPS) generates a continuous raw digital data stream of 7.2–14.4 kbps (assuming 24 bits per axis: 200SPS×3axes×24bits/sample=14.4kbps). However, typical telemetry ingestion protocols, encapsulation overheads (such as MiniSEED formatting), and command interfaces standardize on a physical UART interface speed of 115 200 bps (or 115.2 kbps) at the sensor output. To prevent serial buffer accumulation and ensure immediate transmission, the wireless link must support a sustained goodput (application-level payload throughput) that exceeds the source data rate, preferably matching the serial ingestion speed of 115 200 bps to support packetized burst transmission [2], [3]. Field deployments in southern Peru–including the active Misti and Sabancaya volcanoes monitored by INGEMMET’s Volcano Observatory (OVI) [1]–demand point-to-point radio links over distances of 10–20 km.

The dominant paradigm for low-cost long-range IoT telemetry is LoRa, a proprietary Chirp Spread Spectrum (CSS) modulation operating in sub-GHz ISM bands [4], [5]. Given its exceptional range, LoRa-based projects have become ubiquitous across diverse disciplines, scaling from smart agriculture implementations — such as the PyLoGreen system deployed in the High-Andean Tundra [6] — to multiparametric volcanic monitoring networks [1]. However, the vast majority of existing deployments aim to optimize the physical link layer or energy efficiency (e.g., through custom antennas [7]) while accepting the severe throughput bottlenecks inherent to sub-GHz CSS architectures.

Although LoRa excels in ultra-low-data-rate sensor networks [8], its effective air rate collapses under the high spreading factors required for robust long-distance links: SF10–SF12 yields 1–5 kbps, making CSS fundamentally incapable of sustaining 115 200 bps [9]. This bandwidth constraint becomes critical when transmitting high-density data such as continuous images [10] or real-time waveforms. Furthermore, LoRaWAN’s regional duty-cycle limitations (typically <1% in Europe) [5] and its Adaptive Data Rate (ADR) mechanism [11] are optimized for infrequent payloads, not continuous streams. In dense deployments or continuous transmission scenarios, uncoordinated ALOHA access leads to significant packet collisions, severely limiting network capacity [12]. While attempts have been made to increase LoRa throughput via joint modulation schemes [13], practical sub-GHz implementations still consider rates around 21 kbps as “high speed” [14], far below the requirements of 100 Hz seismic arrays. At the waveform level, recent advanced chirp modulations — such as layered chirp spread spectrum (LCSS) [15] and dual-mode chirp spread spectrum (DM-CSS) [16] — have expanded LPWAN spectral efficiency and capacity by multiplexing chirp structures and phase modes. However, such schemes address a fundamentally different operating point from the sustained, high-rate physical streaming targeted here, where uncompressed continuous scientific streams demand uninterrupted hundreds of kilobits per second.

A direct antecedent is the work of Arana-Medina and Rendulich [7], which empirically validated a 433 MHz LoRa link with a custom Yagi-Uda antenna across the 16 km Misti–Arequipa path. That work confirmed the geometric feasibility of the radio link yet highlighted the fundamental throughput limitation of sub-GHz CSS: the system cannot sustain continuous high-density data streams. Analogous limitations have been documented in other volcanic monitoring deployments [1].

To break this bottleneck, we aim to develop a fully open platform that is as reliable and easy to deploy as traditional LoRa nodes, but engineered explicitly for high-throughput, continuous data streams. We present OpenFLRC, a custom carrier board integrating a Semtech SX1280 transceiver alongside an ESP32-S3 microcontroller. While exploiting the same Semtech silicon ecosystem, our pivotal contribution is granting the user direct bare-metal control over the radio—exposing internal hardware buffers, explicit transmit/receive timings, and direct SPI interrupts via a custom C++ library. This level of deterministic control is not feasible with commercial closed-source LoRa modules, which abstract away the MAC layer and obscure critical packet pacing mechanisms. To empirically validate this architecture, this paper presents a rigorous comparative methodology: we trace Performance Delivery Ratio (PDR) curves of OpenFLRC against two standard sub-GHz LoRa modules under identical laboratory attenuation, evaluating its capability to sustain continuous 115 200 bps telemetry.

The Semtech SX1280/SX1281/SX1282 family [17] integrates three PHY modulations at 2.4 GHz ISM: LoRa, GFSK, and FLRC (*Fast Long-Range Communication*). FLRC at BR_1_300_BW_1_2 achieves 1.3 Mbps air rate with −97 dBm receiver sensitivity–sufficient for multi-kilometre links as demonstrated by Janssen et al. [18] who calculated 133 km free-space range for the SX1280, and by Llap and Nuñez [19] who benchmarked 480 parameter combinations of the SX1280 characterizing energy and maximum distance. Coexistence with Wi-Fi and Bluetooth in the 2.4 GHz band has been studied by Polak and Milos [20], showing that LoRa/FLRC can operate reliably when proper channel selection and duty-cycle management are applied. Müller et al. [21] demonstrated nanosecond-level time synchronization over 170 m using the SX1280 ranging engine–a capability embedded in our hardware for future network expansion [22], [23].

Compared to open-source alternatives, none satisfies all requirements simultaneously. HardwareX publications describe LoRa-based nodes for low-rate environmental monitoring using sub-GHz LoRa [24], [25] and, most recently, an ESP32-S3 sensor with LoRaWAN [26]–which shares our MCU but not the FLRC PHY or the continuous-stream firmware architecture.

Beyond literature-documented systems, several community open-source projects target the SX1280. Low-level C ports such as libsx128x [27] wrap Semtech’s reference C drivers for modular usage, while satellite ground initiatives like the ThingSat tinygs_2g4station [28] demonstrate SX1280 2.4 GHz nodes in orbit-reception environments. However, these platforms are either tailored for low-rate satellite frames or lack PlatformIO integration and user-friendly APIs for continuous stream telemetry, leaving a critical gap that OpenFLRC directly addresses.

Open seismic hardware (such as CEDAS [29] or the open VSP recorder [30]) focuses on acquisition but delegates telemetry to proprietary or low-rate radios. The present work fills this gap with a fully open, reproducible, and characterized 2.4 GHz FLRC transceiver validated against a realistic volcanic monitoring link budget, following open-hardware best practices [31].

### 1.1 Comparative context with existing platforms and physical layers

Table 1 compares OpenFLRC with the most relevant commercial and open-hardware alternatives for continuous high-throughput telemetry. Unlike commercial sub-GHz LoRa modules (E22 series), which embed a closed-firmware MCU acting exclusively as a UART-to-radio bridge, OpenFLRC integrates a fully programmable ESP32-S3 that simultaneously manages the radio and can run user application code—making the $32.70 node a complete computing platform. We also compare against ExpressLRS [32], whose “Airport” transparent telemetry mode saturates at ∼40 kbps due to FHSS framing overhead.

To position OpenFLRC within the broader 2.4 GHz radio landscape, a comprehensive multi-parameter comparison across commercial evaluation boards, bare RF modules, and open firmware implementations (Semtech DVK, Ebyte E28, LILYGO T3S3, ExpressLRS) is provided in Appendix A (Table A.1). Furthermore, a detailed theoretical and empirical comparison across all native SX1280 physical-layer modulations (LoRa, GFSK, and FLRC) and firmware stacks is reported in Table A.2. These analyses confirm that OpenFLRC is the only platform simultaneously delivering +27 dBm output power, sustained 884.3 kbps goodput (PDR≥99.9%) with convolutional forward error correction, and an unencumbered dual-core host architecture for continuous scientific data acquisition.

**Table 1:** Comparison of long-range wireless platforms for 115 200 bps continuous telemetry. CSS: Chirp Spread Spectrum.

Platform	Chip	Band	Mod.	Eff. rate	Onboard MCU	Open HW	Unit cost
E22-900T30D (Ebyte)	SX1262	915 MHz	CSS	∼54 kbps	Closed^c^	No	$12.49
E22-400T30D (Ebyte)	SX1268	433 MHz	CSS	∼35 kbps	Closed^c^	No	$11.69
ExpressLRS Airport mode [32]	SX1280	2.4 GHz	FLRC	∼40 kbps^a^	Partial	Partial	$15–30
**OpenFLRC v1.0 (ours)**	**SX1280**	**2.4 GHz**	**FLRC**	**884.3 kbps**	**ESP32-S3**^b^	**Yes**	**$32.70**

a ExpressLRS Airport saturates at ∼5000 bytes/s due to 64-byte payload buffer limit [17].

b Dual-core Xtensa LX7, 240 MHz, 16 MB flash, 8 MB PSRAM. Runs radio, WiFi/BT, OTA and user application logic simultaneously.

c MCU closed: internal firmware is proprietary; cannot execute user application code.

## 2 Hardware description

The OpenFLRC v1.0 is a 2-layer FR4 carrier board engineered to physically and electrically integrate two commercial modules into a unified, high-reliability telemetry platform: an Espressif ESP32-S3-DevKit-C (Xtensa LX7 dual-core, 240 MHz, 16 MB flash, 8 MB PSRAM) and an Ebyte E28-2G4M27SX RF front-end module (Semtech SX1280 transceiver, Skyworks high-gain PA/LNA delivering up to +27 dBm/500 mW output power, integrated IPEX/U.FL connector, SPI interface).

### Clarification of RF interface and modular boundary.

To maintain rigorous scientific clarity regarding our hardware contributions, we explicitly note that the E28 module integrates its own internal 50 Ω LC impedance-matching network and an onboard IPEX (U.FL) coaxial receptacle. The OpenFLRC carrier board interfaces this port directly to an external bulkhead SMA antenna connector via a short 50 Ω RG178 coaxial pigtail. Consequently, the carrier board deliberately avoids routing unshielded 2.4 GHz microstrip RF traces across the FR4 substrate, thereby eliminating dielectric loss uncertainties and near-field capacitive coupling into adjacent digital buses.

### System-level engineering for continuous-duty scientific operation.

The contribution of the carrier board is not a new RF front-end; it is the system-level engineering required to operate the commercial E28 module as a reproducible, continuous-duty scientific telemetry instrument. While utilizing commercial silicon and module building blocks, the carrier board provides critical electrical and architectural solutions required for sustained high-power continuous transmission that raw breakout boards or standard development kits fail to deliver:

1. **Power Integrity for 550 mA Pulsed Transients:** Driving a +27 dBm power amplifier continuously under high-rate streaming introduces severe electrical stress on the 3.3 V supply rail, drawing rapid square-wave current pulses peaking at 550 mA with nanosecond-scale rise times. From a circuit design perspective, long prototype jumper leads or breadboard contacts introduce parasitic loop inductance ($L_{\mathrm{loop}}\approx 10\text{–}30\;\text{nH/cm}$) that, under fast-edge 550 mA steps, risks severe inductive voltage droop (ΔV=L⋅di/dt) and supply instability. OpenFLRC mitigates this design vulnerability by embedding a specialized 5-capacitor decoupling ladder positioned directly adjacent (<3 mm) to the E28 VCC/GND pins: two 150μF ultra-low-ESR surface-mount tantalum capacitors (providing an immediate local bulk charge reservoir) operating in parallel with three high-frequency X7R ceramic capacitors (10μF, 1μF, and 100 nF). The effectiveness of this physical network is confirmed by uninterrupted, stable transmission without packet rate degradation across all four benchmarking phases (over 180,000 cumulative packet transmissions at 100% duty cycle).
2. **Thermal Dissipation and Field Integration Considerations:** Continuous transmission at ＋27 dBm RF output (∼500 mW RF, ∼1.8 W DC input) results in approximately 1.3 W dissipated as heat, concentrated primarily within the E28 internal power amplifier. Based on the manufacturer hardware documentation [33], the module features a stamped nickel-plated metal shield that acts as a surface radiator. The OpenFLRC carrier board complements this by providing an unbroken 35μm (1 oz) bottom-layer copper ground plane stitched to the module ground pads with thermal vias, functioning as a passive lateral heat spreader. During our laboratory benchmarking sweeps at room temperature (22–25°C), the transceiver operated stably without an active heatsink or observed throughput degradation. While the module temperature noticeably rises under continuous +27 dBm transmission, physical thermal probing was not performed. Designing a dedicated protective enclosure and sizing a tailored heatsink depends directly on the specific mechanical housing and solar/ambient conditions of the deployed field application, which lies beyond the scope of this transceiver platform project. Consequently, incorporating an adhesive aluminum heatsink onto the module shield and providing adequate case ventilation is recommended as part of application-specific field deployment.
3. **Dedicated Physical RF Switch Routing:** The E28 module requires external logical gating of its internal Skyworks RF switches via TX_EN and RX_EN. The carrier board routes these control lines directly through low-capacitance dedicated PCB traces to GPIO 5 and GPIO 6 of the ESP32-S3. This dedicated hardware routing enables the firmware to drive state transitions deterministically within 2.5μs, eliminating RF switch floating states and preventing destructive PA reflections or self-oscillation during transmit-to-receive turnarounds.
4. **Sensor Expansion Breakout for Scientific Instrumentation:** Commercial sub-GHz LoRa modules (e.g., Ebyte E22 series) function strictly as closed, non-programmable serial bridges that forbid user-defined software. In contrast, OpenFLRC is architected as an open edge-computing instrument. The carrier board routes approximately half of the SoC’s uncommitted GPIOs (including dedicated 12-bit SAR ADC channels, high-speed UARTs, and auxiliary I2C/SPI interfaces) to standard 2.54 mm expansion pin headers. This layout allows Core 0 to operate autonomously as a high-precision data acquisition unit—interfacing multi-axis geophones, 24-bit delta-sigma seismic ADCs, or environmental sensors, executing digital FIR filtering, and packaging continuous streams—completely unhindered by RF SPI activity on Core 1.
5. **Scientific Open-Source Reproducibility (HardwareX):** In full alignment with the publication criteria of HardwareX, our contribution is an open-source, fully validated scientific hardware platform. By providing complete production gerbers, editable EasyEDA schematics, a detailed $32.70 USD Bill of Materials, and modular open-source firmware, OpenFLRC enables any research group worldwide to construct and deploy robust high-bandwidth telemetry nodes without relying on costly closed-source industrial telemetry systems.

The ESP32-S3 firmware, written in C++ using the RadioLib library [34] and drawing reference from the open-source SX12XX-LoRa library [35], configures the SX1280 in FLRC mode (FLRC_BR_1_300_BW_1_2): 1.3 Mbps air rate, 1.2 MHz bandwidth, 117-byte maximum payload (127-byte hardware buffer minus 6-byte FLRC header minus 4-byte CRC/NetworkID, per SX1280 datasheet Section 14.4 [17]). The UART↔FLRC bridge is governed by the OpenFLRC firmware and hardware architecture illustrated in Fig. 1. To eliminate the standard serial and radio bottlenecks of existing black-box libraries, OpenFLRC implements a multithreaded task-segmentation scheme under FreeRTOS, dividing operations between the dual-core Xtensa LX7 processor of the ESP32-S3:

1. **Ingestion & Queue Pipeline (Core 0):** A high-priority incoming 115 200 bps UART stream from the geophone digitalizer and stores it in a lock-free circular ring-buffer in RAM, preventing serial frame overrun during RF transactions.
2. **Asynchronous Radio Scheduler (Core 1):** A dedicated thread fetches 117-byte chunks from the ring buffer and pushes them to the SX1280 FIFO over a high-speed SPI bus operating at its physical datasheet limit of 18 MHz with Direct Memory Access (DMA), shrinking SPI payload transit time to ≈56 µs.
3. **Interrupt-Driven State Transitions:** Instead of blocking polling loops, radio state transitions are governed strictly by the DIO1 hardware interrupt, which triggers a RAM-cached Fast Interrupt Service Routine (IRAM_ATTR), instantly resetting the radio to receive or loading the next transmit packet.
4. **MCU-Controlled RF Switching:** To control the half-duplex transmit and receive paths, the host microcontroller directly toggles the module’s external Power Amplifier (TX_EN) via GPIO 5 and the Low Noise Amplifier (RX_EN) via GPIO 6, managed deterministically within the fast state transitions of the radio task.

This highly optimized pipeline achieves a sustained effective goodput of 884.3 kbps (PDR≥99.9%) under conducted laboratory testing, providing a 7.7× margin above the 115 200 bps target.

**Fig. 1:**
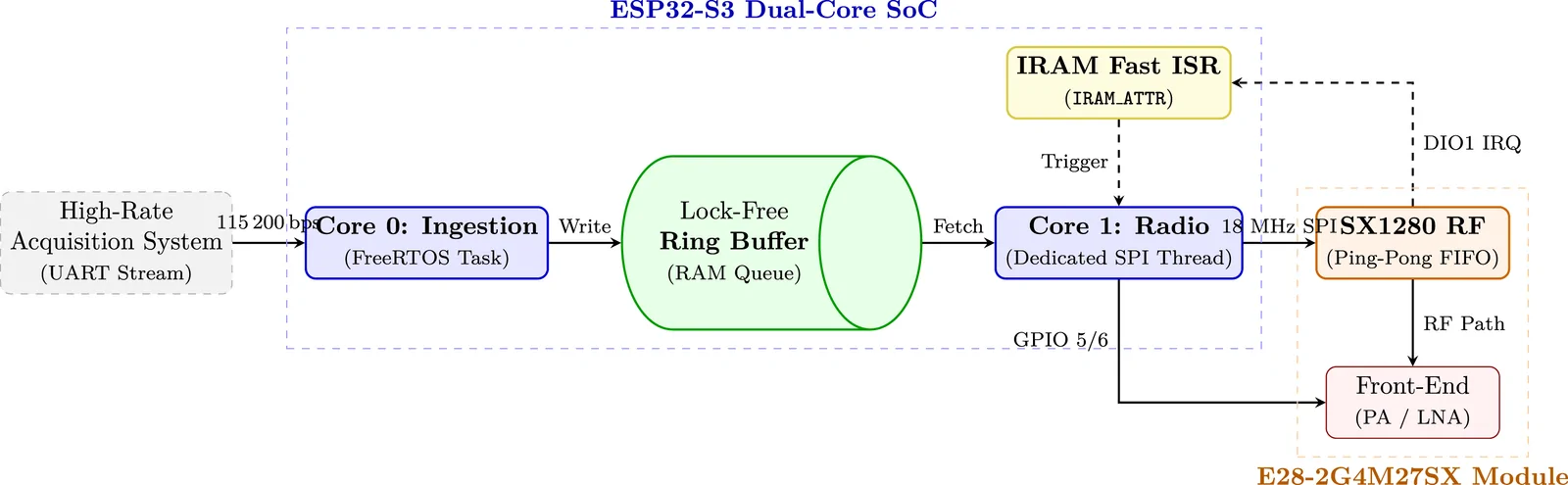
OpenFLRC firmware and hardware architecture. Core 0 handles non-blocking UART stream ingestion into a lock-free circular ring-buffer in RAM. Core 1 runs a dedicated SPI task executing 18 MHz DMA transfers to the SX1280 transception FIFO, while directly driving the external RF Front-End (PA/LNA) via physical GPIO 5 (TX_EN) and GPIO 6 (RX_EN) signaling. Upon packet completion, a hardware interrupt on DIO1 triggers a RAM-cached Fast ISR (IRAM_ATTR) to instantly queue the subsequent transaction.

This open-source transceiver platform is designed to be highly versatile and can help other researchers with a variety of scientific data acquisition and remote monitoring tasks:

• **High-Rate Remote Telemetry:** Enables continuous, real-time wireless transmission of high-density scientific streams (such as multi-channel geophones, vibration sensors, or meteorological arrays) across dedicated point-to-point links without packet loss.
• **Low-Latency Laboratory DAQ:** Provides a low-latency, deterministic wireless link for high-speed in-lab data acquisition, replacing physical cables in space-constrained, rotating, or hazardous experiments.
• **Multi-Node Localization:** Harnesses the SX1280’s built-in time-of-flight ranging engine to support spatial tracking, alignment, and distance estimation of mobile sensor nodes or autonomous field equipment.
• **Open-Source Customizability:** Offers fully accessible C++ firmware and low-level SPI control, allowing researchers to modify transceiver registers, packet sizes, and transmission intervals to match specific experimental needs.
• **Cost-Effective Dedicated Telemetry:** Delivers a high-performance alternative to expensive commercial telemetry systems for only $32.70 USD per node, allowing dedicated high-bandwidth scientific telemetry backbones to be deployed under constrained budgets.

## 3 Design files summary

The OpenFLRC v1.0 hardware platform is officially certified by the Open Source Hardware Association (OSHWA) under UID **PE000005** ( certification.oshwa.org/pe000005.html). All design files are deposited in the Zenodo repository ( doi:10.5281/zenodo.21159006) and include the editable source files compliant with the OSHWA open-source definition [31], as indexed in Table 2.

**Table 2:** Design files summary.

Design filename	File type	Open source license	Location
design_files/OpenFLRC_v1.0_Schematic.json	EasyEDA schematic	CC BY 4.0	Zenodo repository
design_files/OpenFLRC_v1.0_Layout.json	EasyEDA PCB layout	CC BY 4.0	Zenodo repository
design_files/OpenFLRC_v1.0_Gerbers.zip	Gerber + drill (RS-274X)	CC BY 4.0	Zenodo repository
firmware/libraries/OpenFLRC_Library/	C++ abstraction library	CC BY 4.0	Zenodo repository
firmware/benchmarks/	PlatformIO test firmware projects	CC BY 4.0	Zenodo repository
bom.csv	Bill of materials spreadsheet	CC BY 4.0	Zenodo repository
measurements/	Calibration and raw data CSVs	CC BY 4.0	Zenodo repository
videos/phase1_throughput_system_console.mp4	Throughput testing console video	CC BY 4.0	Zenodo repository
videos/phase2_conducted_attenuation_setup.mp4	Conducted attenuation setup video	CC BY 4.0	Zenodo repository
videos/phase3_spectral_validation_tinysa.mp4	TinySA spectral measurement video	CC BY 4.0	Zenodo repository
videos/phase4_antenna_characterisation_vna.mp4	VNA antenna characterization video	CC BY 4.0	Zenodo repository

### 3.1 Design file descriptions

design_files/OpenFLRC_v1.0_Schematic.json / _Layout.json: Full 2-layer carrier board designed in EasyEDA Standard. The schematic includes explicit component values, physical measurements, and connection logic. Routes MOSI, MISO, SCK, NSS (SPI), TX_EN, RX_EN, DIO1 (IRQ), BUSY, and NRST between the ESP32-S3 and the E28-2G4M27SX module. The E28 module’s onboard U.FL port connects directly to the SMA female chassis connector via a 5 cm RG1.13 coaxial pigtail, eliminating the need for high-frequency RF trace routing on the carrier board. An optimized decoupling capacitor ladder, comprising two 150 µF/16 V Case D tantalum capacitors (C1, C2) in parallel as a low-ESR bulk energy reservoir, alongside a 22 µF (C5), a 100 nF (C4), and a 1 nF (C3) 0603 X7R ceramic capacitors, decouples the PA supply rail to suppress the 500 mW TX current surge and filter high-frequency digital and RF noise.

firmware/libraries/OpenFLRC_Library/: Hardware-abstracted C++ library designed for PlatformIO. It handles SPI DMA transactions, pin configurations, and low-level packet transception on the SX1280 transceiver.

firmware/benchmarks/: Contains PlatformIO C++ firmware projects for all testing phases:

• sx1280_throughput_system/, lora_915_throughput_system/, lora_433_throughput_system/: Dynamic Load (Phase 1) benchmark code.
• sx1280_attenuation_sweep/, lora_915_attenuation_sweep/, lora_433_attenuation_sweep/: Conducted Path-Loss (Phase 2) sweep code.
• sx1280_spectrum_test/: CW and modulated test code for Spectral Validation (Phase 3).
• sx1280_diagnostic/: General hardware diagnostic utility for startup validation.

measurements/: Holds all Touchstone S-parameter files (.s1p) captured from the NanoVNA for the attenuator and antennas calibration, as well as CSV outputs from the dynamic load interval sweeps, conducted path loss sweeps, occupied bandwidth, and harmonic sweeps.

## 4 Bill of materials summary

The complete BOM is provided in bom.csv (Zenodo). All components were purchased from AliExpress; real paid prices are listed below (coupons and discounts applied). JLCPCB is used for PCB fabrication. The per-node transceiver component costs are detailed in Table 3, and the one-time shared laboratory benchmark and characterization equipment costs are itemized in Table 4.

**Table 3:** Bill of materials for a single OpenFLRC transceiver node (TX or RX unit).

Desig.	Component	Qty	Cost (USD)		Source	Material type
			Unit	Total		
U1	ESP32-S3-DevKit-C (N16R8, 16 MB flash, 8 MB PSRAM, 240 MHz)	1	8.18	8.18	AliExpress	Semiconductor
RF1	Ebyte E28-2G4M27SX (SX1280, 2.4 GHz, 27 dBm, IPEX, 15 × 20 mm)	1	6.71	6.71	AliExpress	Semiconductor
PCB	Carrier board FR4 2-layer, 100 × 80 mm (JLCPCB, min. qty 5)	1	8.00	8.00	JLCPCB	Composite
C1, C2	Tantalum capacitor 150 µF /16 V, Case D (Bulk decoupling)	2	0.40	0.80	AliExpress	Semiconductor
C3–C5	Ceramic capacitors 0603 X7R (1 nF , 100 nF , 22 µF )	3	0.05	0.15	AliExpress	Ceramic
U2	Buck converter MP1584EN module, 5 V/3 A	1	1.02	1.02	AliExpress	Semiconductor
J1	SMA female chassis connector, PCB-mount, 50 Ω	1	0.40	0.40	AliExpress	Metal
W1	Pigtail SMA-K to U.FL/IPEX, 5 cm, RG1.13 coaxial	1	0.68	0.68	AliExpress	Composite
ANT1	Omnidirectional antenna 2.4 GHz, 10 dBi, SMA Male, 200 mm	1	6.76	6.76	AliExpress	Non-specific

**Total per transceiver node (TX or RX)**				**32.70**

**Table 4:** Benchmark and characterization equipment (one-time shared cost).

Desig.	Component	Qty	Cost (USD)		Source	Material type
			Unit	Total		
VNA	NanoVNA-F V3, 1 MHz–6 GHz, S11/S21/VSWR/Smith	1	165.85	165.85	AliExpress	Semiconductor
SA	TinySA Ultra spectrum analyzer, DC–5.3 GHz	1	111.27	111.27	AliExpress	Semiconductor
ATT1–4	Fixed SMA attenuator 30 dB/2 W, DC–6 GHz (Au-plated)	4	5.19	20.76	AliExpress	Metal
ATT5–6	Fixed SMA attenuator 10 dB/2 W, DC–6 GHz	2	7.62	15.24	AliExpress	Metal
ATT7	Fixed SMA attenuator 5 dB/2 W, DC–6 GHz	1	5.32	5.32	AliExpress	Metal
CBL	Pigtail SMA M-M RG316, pack of 5 units	1	13.79	13.79	AliExpress	Composite
OSL	SMA OSL calibration kit for VNA	1	4.18	4.18	AliExpress	Metal
DL	Dummy load SMA 50 Ω/2 W, pack of 2	1	1.29	1.29	AliExpress	Metal
REF1–2	Benchmark: Ebyte E22-400T30D (SX1262, 433 MHz, 30 dBm, UART)	2	11.69	23.38	AliExpress	Semiconductor
REF3–4	Benchmark: Ebyte E22-900T30D (SX1262, 915 MHz, 30 dBm, UART)	2	12.49	24.98	AliExpress	Semiconductor
ANT2–5	Omnidirectional antenna 2.4 GHz, 10 dBi, SMA (pack of 4)	4	6.76	27.02	AliExpress	Non-specific
ANT6–7	Flexible antenna 433 MHz, 10 dBi, pack of 2	1	–	10.63	AliExpress	Non-specific
ANT8–9	Flexible antenna 915 MHz, 10 dBi, pack of 2	1	–	10.63	AliExpress	Non-specific

**Total characterization equipment (real paid prices)**				**434.33**		

**Complete project total (hardware acquired)**				**501.94**		

## 5 Build instructions

### 5.1 Step 1 – PCB fabrication

Export Gerber files from the EasyEDA Standard project layout files (flrc_node_v1_layout.json) and order from a PCB service (e.g., JLCPCB). Recommended specifications for the PCB substrate and manufacturing are: FR4, 2 layers, 1.6 mm board thickness, HASL lead-free surface finish, and a minimum track/space width of 0.15 mm. The fabrication cost is extremely low (≈$2.10 USD for 5 units), though the total acquisition price depends heavily on the destination region and selected shipping method. For instance, express delivery via FedEx International Priority to South America (Arequipa, Peru) incurs a shipping fee of $34.55 USD, yielding a total cost of $26.65 USD after applying standard new-user promotional discounts (−$10.00 USD).

#### 5.1.1 Schematic design and microcontroller pin allocation

The full schematic of the transceiver node is presented in Fig. 2. To maximize system expandability and ensure the user has complete access to the microcontroller’s hardware resources, the routing connects to only one side of the ESP32-S3 DevKit-C pin headers. This leaves the entire opposite side of the ESP32-S3 module’s pinout completely free and unconnected, allowing the end user to interface other digital or analog sensors, logging modules, or actuators directly to the node.

Additionally, the digital pins DIO2 and DIO3 of the Semtech SX1280 transceiver are left unconnected (floating) to permit custom user hardware expansion and configuration options.

**Fig. 2:**
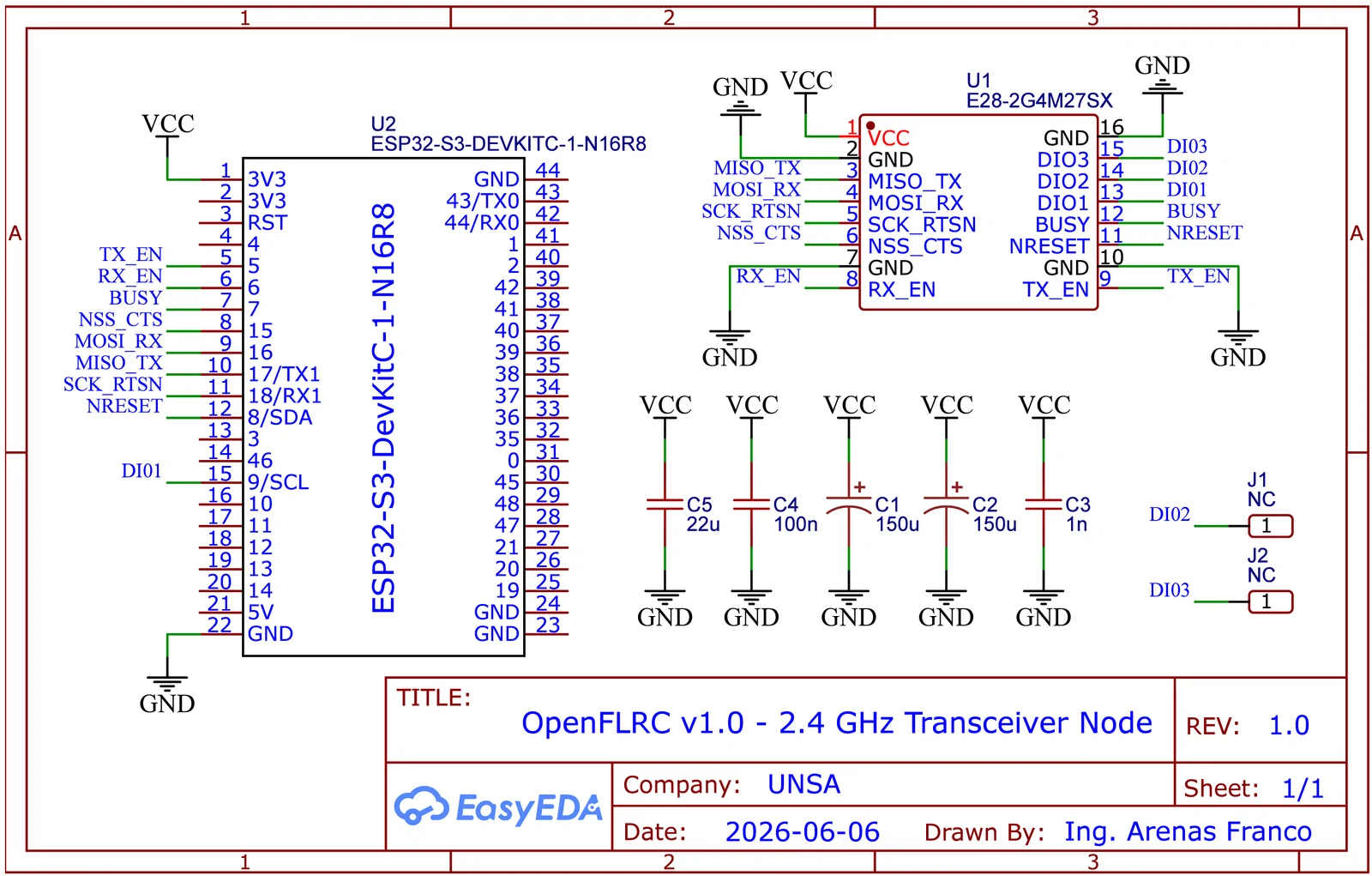
Schematic diagram of the OpenFLRC v1.0 carrier board. Only one side of the ESP32-S3 module is routed, leaving the opposite side fully free for user extensions. The decoupling capacitor ladder (C1–C5) stabilizes the power rail during RF transmit spikes, and pins DIO2/DIO3 are left unconnected for custom configurations.

#### 5.1.2 RF interface and impedance matching

To avoid high-frequency attenuation, dielectric losses, and impedance mismatches associated with routing 2.4 GHz RF signals on a standard 2-layer FR4 substrate, the carrier board does not route any RF copper traces. Instead, the Ebyte E28-2G4M27SX transceiver module features an onboard pre-matched 50 Ω U.FL (IPEX) connector. A pre-fabricated 50 Ω characteristic impedance RG1.13 coaxial pigtail cable (5 cm length, U.FL to SMA female) is connected directly from the module to the chassis-mounted SMA connector. This ensures impedance matching across the RF path to the external 50 Ω omnidirectional antenna, keeping insertion losses to a minimum.

#### 5.1.3 Bulk power decoupling network

To handle the high transient current surges during radio transmission (＋27 dBm/500 mW), the carrier board incorporates two 150 µF/16 V Case D tantalum capacitors in parallel (C1 and C2) acting as a low-ESR bulk energy reservoir to prevent supply voltage drops. High-frequency digital and RF noise is filtered by a decoupling capacitor ladder placed adjacent to the E28 module footprint, consisting of a 22 µF (C5), a 100 nF (C4), and a 1 nF (C3) 0603 X7R ceramic capacitors.

#### 5.1.4 PCB layout geometry and design rules

For the physical PCB layout design, specific design rules are applied to manage power and signal paths. Power routing nets (such as GND and VCC lines) are designed with a track width of 1.0 mm and a minimum clearance of 0.2 mm to safely handle transient transmission current spikes. Default signal nets are routed using a standard track width of 0.254 mm (10 mil) and a minimum clearance of 0.152 mm (6 mil). Detailed top and bottom copper layout views are shown in Fig. 3.

The final 3D representations of both the top and bottom assembled layers are shown in Fig. 4, displaying the physical component layout and the overall form factor of the module.

**Fig. 3:**
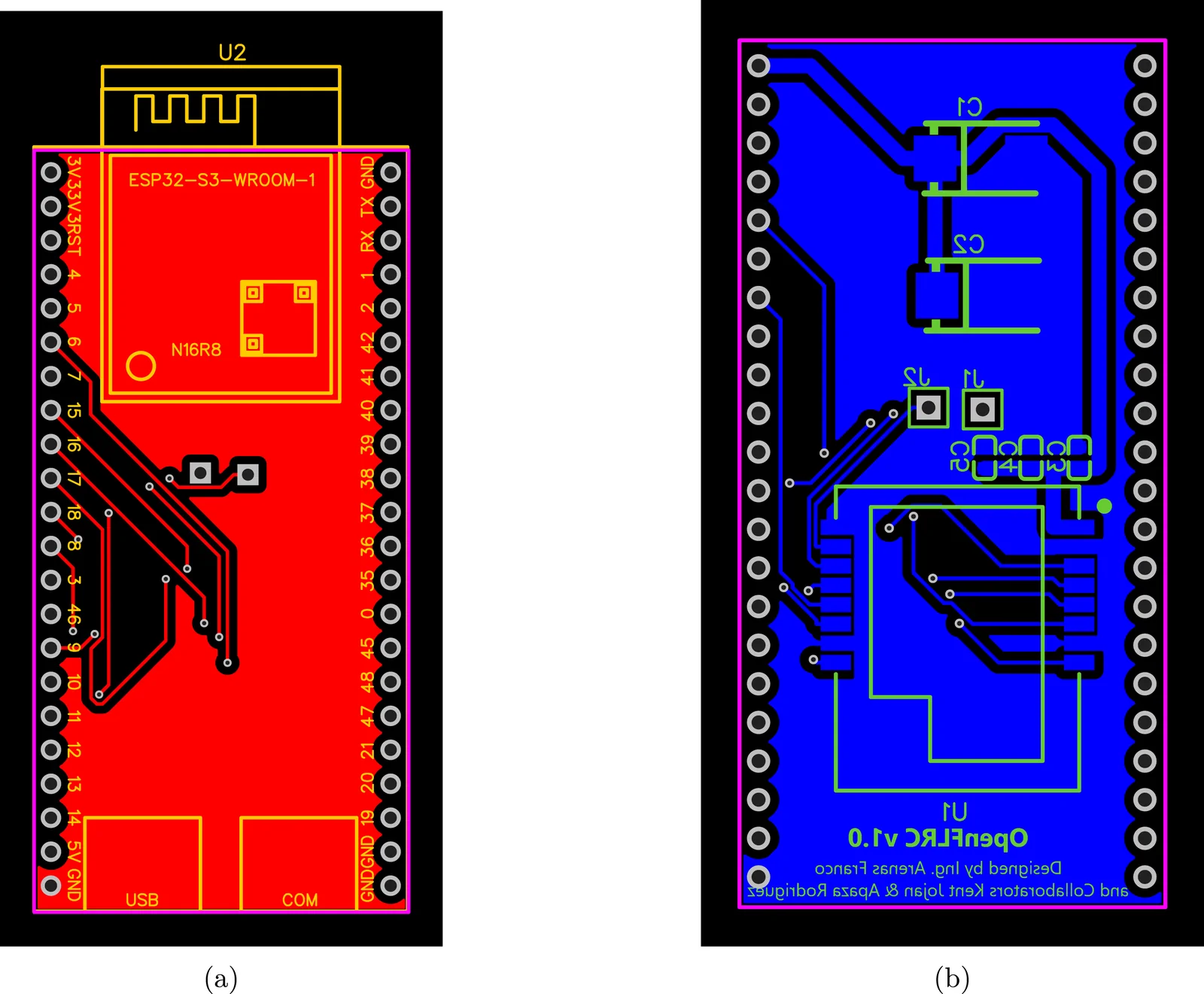
PCB copper layout and silkscreen prints of the OpenFLRC v1.0 transceiver board: (a) top layer, (b) bottom layer.

**Fig. 4:**
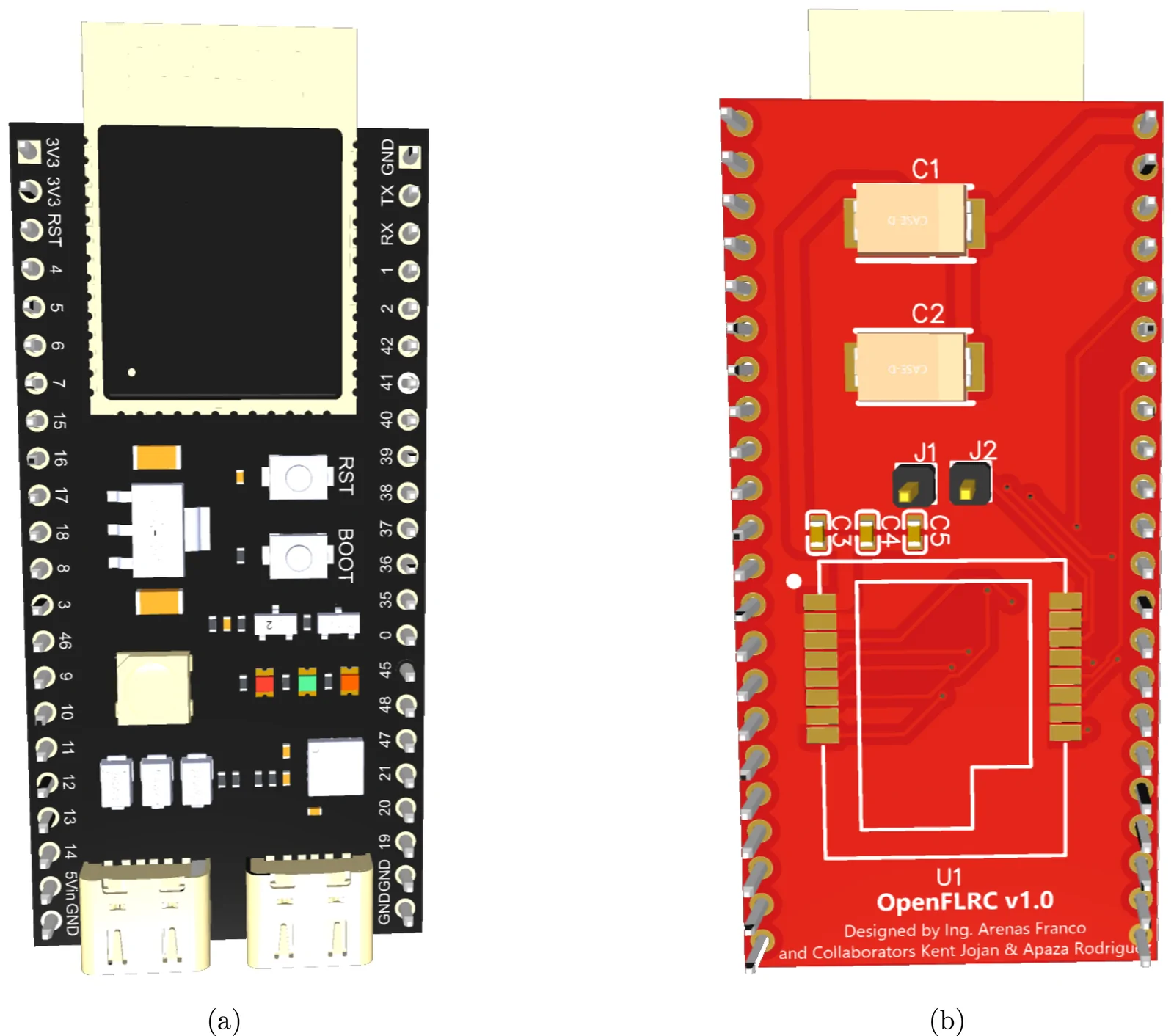
3D visual renders of the OpenFLRC v1.0 carrier board: (a) top side showing the ESP32-S3 DevKit module, USB-C ports, and buttons, (b) bottom side showing the E28 transceiver module, decoupling tantalum capacitors, and silkscreen credits.

### 5.2 Step 2 – Component assembly

Assemble in the following order to avoid mechanical interference:

1. Solder the SMA chassis connector (J1); verify 50 Ω continuity with a multimeter.
2. Solder the decoupling capacitor ladder adjacent to the E28 module footprint: the two 150 µF/16 V Case D tantalum capacitors (C1, C2, observing polarity), and the three 0603 ceramic capacitors (C3–C5, placing the 1 nF capacitor C3 closest to the VCC pin).
3. Seat the Ebyte E28-2G4M27SX module (RF1) in its through-hole footprint and solder all pins. Attach the U.FL-to-SMA pigtail (W1) **before** powering on.
4. Seat the ESP32-S3-DevKit-C (U1) in the dual-row 2.54 mm header sockets.

The physical outcome of the assembly procedure is shown in Fig. 5, displaying the completed carrier board alongside the experimental conducted bench configuration.

**Safety:** *Never* power the E28 module without a 50 Ω antenna or dummy load on the SMA port. The SX1280 PA outputs up to 500 mW (＋27 dBm) at 2.4 GHz; operating into an open port will damage the amplifier stage [33], [36].

**Fig. 5:**
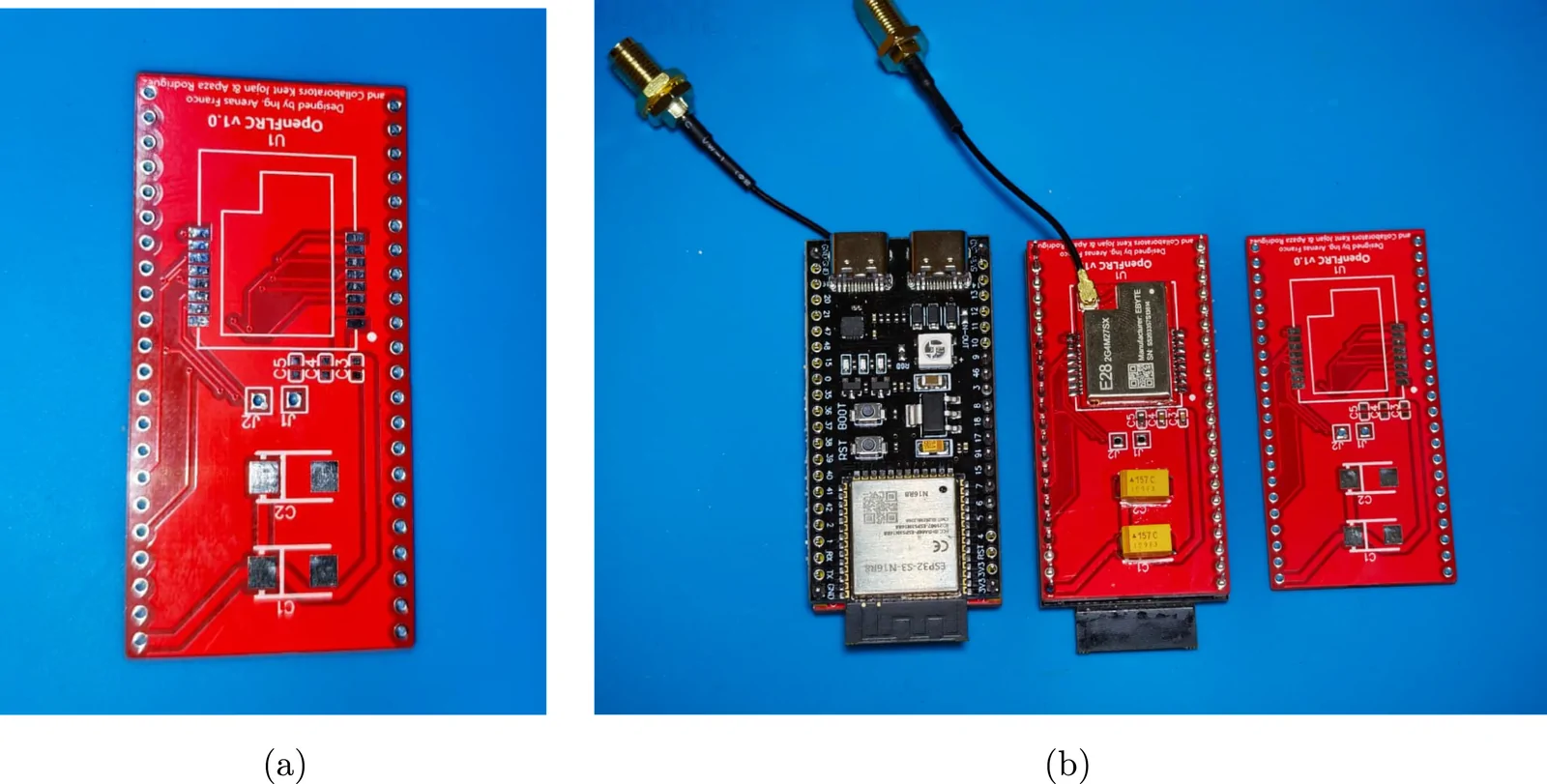
Assembled OpenFLRC v1.0 transceiver hardware: (a) close-up of the fully assembled PCB with Ebyte E28 module, decoupling tantalum capacitors, and headers populated, and (b) physical laboratory test setup showing the master transceiver connected to the coaxial RF conducted attenuation cabling.

### 5.3 Step 3 – Firmware architecture and flashing

The firmware for the OpenFLRC platform is architected in two distinct layers: a hardware-abstracted C++ library (OpenFLRC_Library) and the end-user application.

The OpenFLRC_Library is a fully open-source and free modification that provides a robust, object-oriented abstraction. It builds upon the foundational RadioLib API [34] and draws architectural inspiration from the SX12XX-LoRa repository [35], but it is specifically optimized for the bare-metal capabilities of the Semtech SX1280 [17] and the Ebyte E28-2G4M27SX hardware frontend [33]. It handles low-level hardware initialization, deterministic SPI transactions using DMA (Direct Memory Access) allocations (heap_caps_malloc(..., MALLOC_CAP_DMA)), and sub-millisecond polling of the BUSY and DIO1 pins to bypass FreeRTOS context-switching latencies. This library is entirely application-agnostic and can be seamlessly integrated into any ESP32 project requiring high-speed FLRC links.

For the purpose of validating this hardware, the repository includes a specific application: the **Throughput Benchmark System** (sx1280_throughput_system). This application builds on top of the OpenFLRC_Library to implement a bidirectional polling state machine, a Wi-Fi asynchronous web server for remote test control, and Over-The-Air (OTA) flashing capabilities.

To compile and flash the throughput benchmark application:

1. Install PlatformIO IDE (VS Code extension) with the espressif32 platform package.
2. Download and extract the Zenodo archive, then open the firmware/benchmarks/sx1280_throughput_system/ project folder in PlatformIO.
3. Verify that the platformio.ini resolves the local OpenFLRC_Library dependency correctly.
4. In src/main.cpp, configure the node role by setting #define IS_MASTER true (for the transmitter) or false (for the receiver).
5. Connect the ESP32-S3-DevKit-C via USB-C; select the correct COM port and the esp32-s3-devkitc-1 environment.
6. Execute pio run --target upload. Open the serial monitor at 115 200 bps and verify the startup message indicating the successful initialization of the E28-2G4M27SX module.

**Table 5:** SPI and control GPIO assignments – prototype wiring (YD-ESP32-S3, left-side header). All GPIOs verified against VCC-GND Studio Mischianti pinout. **Note:** GPIO 35, 36, 37 are unavailable on the N16R8 variant (occupied by PSRAM/Flash).

Signal	GPIO	Description
NSS/CS	15	SPI chip-select for SX1280 (active-low)
SCK	18	SPI clock
MOSI	16	SPI data out (ESP32 → SX1280)
MISO	17	SPI data in (SX1280 → ESP32)
BUSY	7	SX1280 busy flag (poll before each SPI transaction)
DIO1	9	SX1280 IRQ – packet done/RX timeout
TX_EN	5	PA enable – active-high, driven by ESP32-S3 (PA active high)
RX_EN	6	LNA enable – active-high, driven by ESP32-S3 (LNA active high)
DIO2	N/C (−1)	Unconnected (floating)
DIO3	N/C (−1)	Unconnected (floating)
NRST	8	SX1280 hardware reset (active-low, 50 kΩ pull-up)

## 6 Operation instructions

### 6.1 Importing the OpenFLRC library into a custom project

While Zenodo hosts the immutable publication snapshot of the OpenFLRC repository to ensure long-term scientific reproducibility, active development and issue tracking occur on GitHub. To utilize the hardware in a custom telemetric application, the user should import the library into a new PlatformIO project:

1. Initialize a new PlatformIO project for the esp32-s3-devkitc-1 board.
2. Clone the OpenFLRC library directly from GitHub into the lib/ directory of the project: cd lib/ && git clone https://github.com/FrancoArenasM/OpenFLRC_Library.git
3. In the platformio.ini file, configure the dependencies to include the core RadioLib API: lib_deps = jgromes/RadioLib @ ^7.1.2
4. In the user application (main.cpp), instantiate the OpenFLRC object, configure the SPI pins according to Table 5, and call the initialization method. The radio is now ready to transmit or receive high-throughput FLRC payloads.

### 6.2 Running the throughput benchmark application

The following instructions detail how to operate the included **Throughput Benchmark System** application, which was used to validate the hardware in this paper.

1. Power both TX and RX nodes via USB-C (5 V) or an external 3.3 V supply connected directly to the capacitor bank logic (bypassing USB regulation).
2. Access the ESP32-S3 Wi-Fi access point (e.g., sx1280-master-tp) and navigate to the asynchronous web server at http://192.168.15.14 to view the testing console.
3. From the web interface, the user can trigger automated bidirectional ping sweeps that decrease the inter-packet interval down to 150 µs, logging the RSSI, SNR, and exact DMA-measured microsecond inter-arrival times.
4. Download the resulting CSV metrics directly from the RX node’s web console for post-processing.

## 7 Validation and characterization

**Safety considerations:**

• Always terminate unused RF ports with a 50 Ω dummy load *before* powering the TX node.
• The E28-2G4M27SX PA radiates at +27 dBm (500 mW) at 2.4 GHz. Maintain appropriate separation or use conducted measurements only. Comply with local 2.4 GHz ISM band regulations [5], [36].
• Handle the SX1280 module with anti-static precautions. The RF front-end is sensitive to electrostatic discharge (ESD) [33].

### 7.1 Conducted-RF comparative benchmarking methodology

To rigorously validate the OpenFLRC platform, we designed a three-radio comparative benchmarking protocol that evaluates OpenFLRC against the two dominant sub-GHz LoRa alternatives under identical, reproducible laboratory conditions. This section describes the experimental setup, the instrumentation chain, the firmware test protocol, and the metrics captured.

#### 7.1.1 Radios under test

The benchmarking campaign compares the custom OpenFLRC platform (E28-2G4M27SX transceiver in FLRC mode) against the two dominant sub-GHz LoRa modules (E22-400T30D at 433 MHz and E22-900T30D at 915 MHz) described in Section 1.1. All three modules are driven by identical host microcontrollers. While the sub-GHz modules communicate via a legacy UART interface, the OpenFLRC module utilizes a direct SPI bus with explicit TX_EN/RX_EN half-duplex control of the integrated Front-End Module (FEM). The key RF parameters under test (configured output power and receiver sensitivity) are detailed in the subsequent link budget analysis (Table B.1 in Appendix B).

#### 7.1.2 Conducted-RF test bench

All measurements across the three bands are performed as *conducted* (wired) RF tests using identical coaxial cabling (consistent with the VNA calibration) and two series 30 dB attenuators (completely omitting antennas), thereby eliminating air propagation delay, multipath fading, antenna pattern irregularities, and environmental interference [37]. The physical topology is illustrated in the block diagram below:

The attenuator chain consists of fixed 50 Ω SMA attenuators rated at 2 W, DC–6 GHz (gold-plated): 4 × 30 dB + 2 × 10 dB + 1 × 5 dB = **145 dB maximum** in discrete 5 dB steps. This wide dynamic range allows the test bench to simulate the free-space path loss (FSPL) of various long-distance geographic locations. For instance, it exceeds the worst-case FSPL of a 16.4 km volcanic telemetry scenario (116.5 dB at 2.44 GHz, detailed in Appendix B) by 28.5 dB, providing a conservative safety margin for characterizing ultra-long-range links (see Fig. 6).

**Fig. 6:**
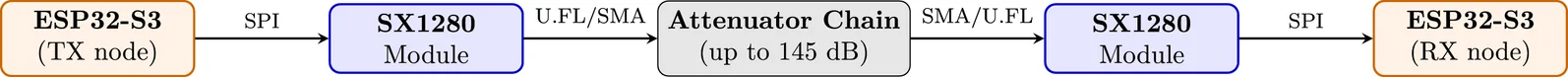
Block diagram of the conducted-RF test bench topology.

#### 7.1.3 Instrumentation

Two portable instruments are integrated into the measurement workflow:

1. **NanoVNA-F V3** (1 MHz–6 GHz vector network analyzer) [38]: Used to perform SOLT (Short-Open-Load-Through) calibration and measure the exact S21 insertion loss of the complete attenuator chain at each radio’s operating frequency (433 MHz, 915 MHz, 2.44 GHz). This eliminates the uncertainty introduced by connector tolerances and cable losses, providing the true attenuation value for link-budget calculations. Additionally, S11 (return loss) and VSWR of the omnidirectional antennas are measured to verify impedance matching at each band.
2. **TinySA Ultra** (DC–5.3 GHz spectrum analyzer) [39]: Used to verify the actual TX output power of each module and to measure the occupied bandwidth (OBW) of each modulation scheme. A minimum of 30 dB (SX1280) or 40 dB (E22) attenuation is always inserted between the TX port and the TinySA input to protect the instrument (maximum safe input: +10 dBm). The real TX power is computed as: $P_{\mathrm{TX}}=P_{\mathrm{TinySA}}+{\left|S_{21}\right|}_{\mathrm{VNA}}$.

#### 7.1.4 RF attenuator chain calibration

To ensure absolute precision and safety during conducted testing, the RF attenuation chain must be rigorously characterized. Because the total nominal attenuation (145 dB) far exceeds the dynamic range of the NanoVNA-F V3 (65 dB up to 3 GHz [38]), a direct single-shot measurement will only register the instrument’s noise floor. To overcome this limitation, the chain was characterized **segment-by-segment** at the central operating frequencies of the three radios: 433 MHz, 915 MHz, and 2.44 GHz.

1. Each of the four nominal 30 dB attenuator pads, the two nominal 10 dB pads, and the single nominal 5 dB pad were measured individually through the OSL-calibrated THROUGH port.
2. S21 (insertion loss) and S11 (return loss) traces were captured for each individual pad at each target band (see Fig. 7). The empirical results of this 3-band calibration are detailed in Table 6.
3. The total conducted path loss of the complete chain is mathematically calculated by algebraically summing the measured S21 values of each individual segment for each frequency:
  (1) $$S_{21,\mathrm{total}}^{\;433\;\mathrm{MHz}}=\overset{7}{\underset{i=1}{\sum }}S_{21,i}=-145.68\;\mathrm{dB}\;\left(\Delta =-0.68\;\mathrm{dB}\right)$$
  (2) $$S_{21,\mathrm{total}}^{\;915\;\mathrm{MHz}}=\overset{7}{\underset{i=1}{\sum }}S_{21,i}=-145.18\;\mathrm{dB}\;\left(\Delta =-0.18\;\mathrm{dB}\right)$$
  (3) $$S_{21,\mathrm{total}}^{\;2.44\;\mathrm{GHz}}=\overset{7}{\underset{i=1}{\sum }}S_{21,i}=-145.34\;\mathrm{dB}\;\left(\Delta =-0.34\;\mathrm{dB}\right)$$
  This demonstrates a high physical accuracy with a deviation of only 0.68 dB at 433 MHz, 0.18 dB at 915 MHz, and 0.34 dB at 2.44 GHz from the nominal 145 dB target. These values establish the true baseline path attenuation for the subsequent performance validation tests. To ensure full reproducibility, the raw NanoVNA calibration state files and their associated Touchstone (.s1p) trace data for each frequency band are publicly hosted in the project’s Zenodo repository.
4. Confirm the TX power of each radio module using the TinySA Ultra [39]:
  • For the E28 module (2.44 GHz): Connect the TX port through a 30 dB attenuator pad (e.g., Pad 1) to the TinySA input. Enable SX1280 CW mode and confirm ＋27 dBm ± 1 dB.
  • For the E22-900T30D module (915 MHz): Connect the TX port through a 30 dB attenuator pad (e.g., Pad 1) to protect the TinySA input. Enable CW mode and confirm ＋30 dBm ± 1 dB.
  • For the E22-400T30D module (433 MHz): Connect through a 30 dB attenuator pad (e.g., Pad 1); confirm ＋30 dBm ± 1 dB.

**Table 6:** Empirical calibration of the SMA attenuator chain at the three target operating frequencies (433 MHz, 915 MHz, and 2.44 GHz) using the NanoVNA-F V3. The total conducted attenuation is computed via segment-by-segment algebraic summation to overcome the VNA’s noise-floor limitations.

Pad ID (Nominal)	433 MHz			915 MHz			2.44 GHz		
	$S_{21}$ (dB)	$S_{11}$ (dB)	RL (dB)	$S_{21}$ (dB)	$S_{11}$ (dB)	RL (dB)	$S_{21}$ (dB)	$S_{11}$ (dB)	RL (dB)
Pad 1 (30 dB )	−29.89	−30.57	30.57	−30.56	−34.90	34.90	−29.74	−20.02	20.02
Pad 2 (30 dB )	−30.84	−31.13	31.13	−29.80	−25.54	25.54	−30.43	−24.96	24.96
Pad 3 (30 dB )	−30.56	−31.65	31.65	−29.83	−19.84	19.84	−29.89	−19.45	19.45
Pad 4 (30 dB )	−29.87	−29.14	29.14	−30.74	−36.43	36.43	−30.67	−25.99	25.99
Pad 5 (10 dB )	−9.68	−33.07	33.07	−9.57	−31.96	31.96	−9.70	−23.72	23.72
Pad 6 (10 dB )	−9.79	−32.46	32.46	−9.57	−30.10	30.10	−9.84	−22.11	22.11
Pad 7 (5 dB )	−5.05	−34.85	34.85	−5.11	−28.10	28.10	−5.07	−22.42	22.42

**Total chain (145 dB )**	**−145.68**	**—**	**—**	**−145.18**	**—**	**—**	**−145.34**	**—**	**—**

**Note:** Measurements performed after SOLT calibration. Instrument frequency accuracy: ±1 ppm. S21 dynamic range: 65 dB.

**Fig. 7:**
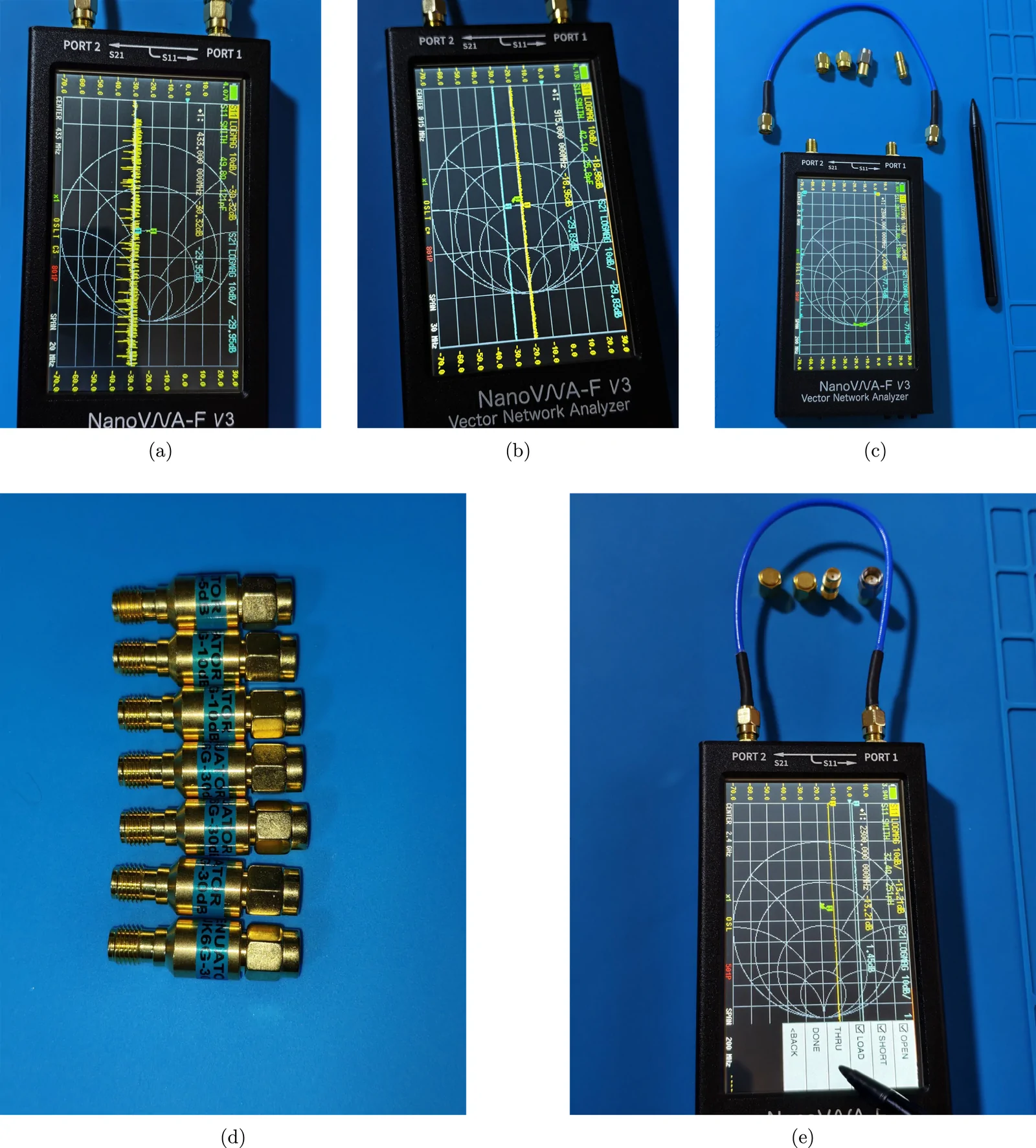
NanoVNA-F V3 RF attenuator chain characterization setup. Top row (a–c) displays the segment-by-segment $S_{21}$ measurements at 433 MHz, 915 MHz, and 2.44 GHz respectively. Bottom row (d–e) details the connection methodology for testing the insertion loss of specific attenuator combinations (e.g., series 30 dB pads) to construct the precise nominal 145 dB baseline.

#### 7.1.5 Firmware test protocol

A unified firmware test protocol ensures a fair comparison across all three radios, structured as a multi-phase validation campaign. In the first phase (Phase 1: Dynamic Load testing), we evaluate the processing latency and packet turnaround limitations under identical, constant attenuation to isolate firmware, SPI, and microcontroller bottlenecks from RF channel degradation. Specifically, all three radios (433 MHz, 915 MHz, and 2.4 GHz) were benchmarked under strict equality of conditions using the exact same ESP32 host microcontroller, running with the asynchronous Wi-Fi stack and Over-The-Air (OTA) flashing features active. This setup replicates a realistic, load-intensive scientific monitoring scenario. To ensure identical path loss conditions, all three configurations used two calibrated 30 dB SMA attenuators connected in series (totaling 60 dB of nominal conducted attenuation) and the exact same coaxial cables calibrated during the VNA baseline. Every test burst was executed using the same transaction state machine logic, and the complete source code for these firmware test scripts is open and available in our database repository.

The protocol systematically sweeps the programmed inter-packet interval under the maximum supported payload size (MTU) of each technology, isolating processing limits under maximum load:

1. **Sub-GHz LoRa (433 MHz and 915 MHz):** Evaluated using a fixed payload size of **240 bytes** (the E22 module’s serial MTU limit), progressively stepping down the programmed interval through 150 ms, 100 ms, 50 ms, 30 ms, 20 ms, 10 ms, 2 ms, and 0 ms to identify the throughput saturation point.
2. **OpenFLRC 2.4 GHz:** Evaluated using a fixed payload size of **117 bytes** (the SX1280 FLRC packet configuration limit), progressively stepping down the programmed interval through 150 ms, 100 ms, 50 ms, 30 ms, 20 ms, 10 ms, 8 ms, 6 ms, 4 ms, 2 ms, and 0 ms to map the limits of the timing loops.

Regardless of the radio module under test, each test burst consists of a predetermined sequence of data packets (e.g., 100 or 1000 packets per burst). Each packet payload encapsulates a structured header containing a unique *Test ID*, a monotonic *Sequence Number*, and a *Transmission Timestamp*, with the remainder of the payload padded to reach the target byte size. The embedded sequence numbers allow the receiving node to detect missing packets and calculate the precise Packet Delivery Ratio (PDR).

For the OpenFLRC platform, this entire benchmarking protocol is orchestrated automatically by the sx1280_throughput_ system application. The master node utilizes an asynchronous Wi-Fi web server to accept test parameters (packet count, payload size, interval) via a web interface, initiates the transmission burst, and collects statistics from the slave node. The derived per-packet metrics — including RSSI, SNR*, transit time, and instantaneous throughput — are aggregated in the ESP32’s memory and exported directly as a consolidated CSV file for post-processing, removing the need for an external serial data logger.

*SNR is natively available from the SX1280 hardware (via RadioLib getSNR()) but not from the sub-GHz E22 UART modules, which only provide a raw RSSI byte.

#### 7.1.6 Attenuation-sweep procedure

The second phase (Phase 2: Conducted Path-Loss Sweep) represents the core experiment, consisting of a systematic *PDR vs. attenuation* sweep performed identically for each radio. To guarantee full replicability and account for connector tolerances, the nominal 5 dB steps are achieved by systematically combining the seven calibrated SMA attenuator pads as detailed in Table C.1 (see Appendix C). The real physical attenuation is calculated by summing the individual S21 values measured by the NanoVNA-F V3 at each frequency band. The physical deployment of the coaxial attenuation chain under the maximum nominal attenuation path loss (145 dB) is pictured in Fig. 8, demonstrating the connection interface between the transceiver nodes.

The sweep procedure is executed as follows:

**Fig. 8:**
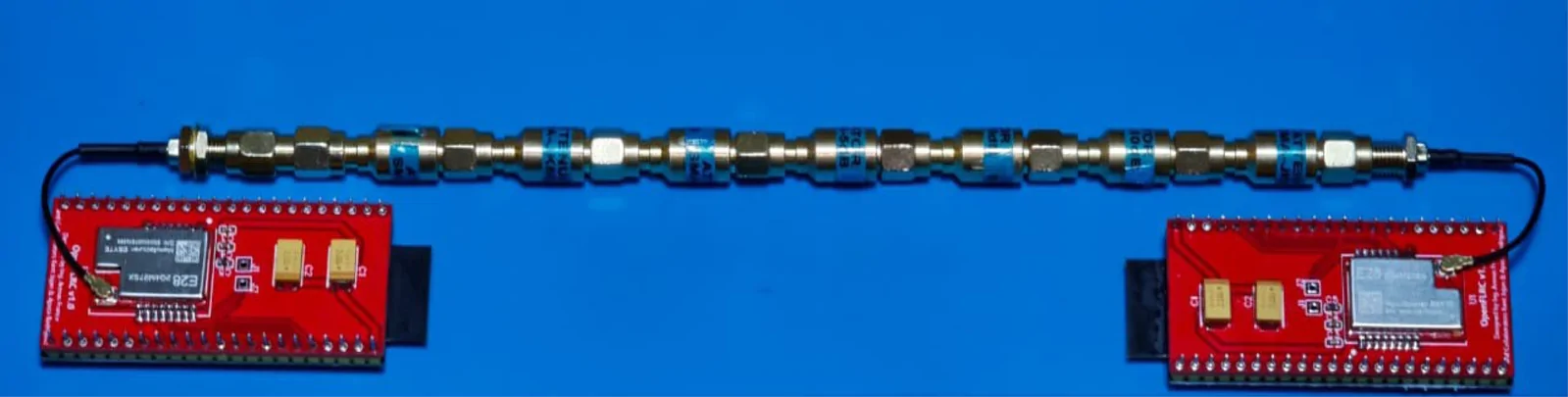
Physical conducted RF benchmarking setup displaying the maximum attenuation configuration (nominal 145 dB path loss). The chain integrates multiple series-connected SMA attenuator pads (30 dB, 10 dB, and 5 dB) calibrated via the NanoVNA, connecting the master node transmitter RF output port to the slave receiver input port inside a shielded laboratory environment to isolate the wireless link.

1. Initialize the transmitter (TX) and receiver (RX) nodes using the most stable, high-speed configuration (payload size and interval) identified in Phase 1.
2. Begin with the minimum safe attenuation of 30 dB (Pad 1) to protect the receiver’s Low Noise Amplifier (LNA) from RF saturation.
3. At each attenuation step, transmit a burst of 1000 packets (10 × 100-packet bursts) and record the PDR (%), average RSSI (dBm), SNR (dB, where available), jitter (ms), and effective throughput (kbps).
4. Systematically increase the conducted path loss in 5 dB nominal increments by connecting the corresponding pads (Table C.1 in Appendix C).
5. Continue the sweep until the PDR drops to 0%, defining the empirical receiver sensitivity threshold for each band.

Additionally, in the third phase (Phase 3: Spectral Validation), we connect the active transmitter’s RF port directly to the TinySA Ultra spectrum analyzer (using the required 30 dB safety attenuation) during the maximum-throughput transmissions. We configure the spectrum analyzer to capture the occupied bandwidth (OBW) and verify that the physical signal is free from out-of-band harmonics or spurious noise, validating the RF front-end’s spectral efficiency and compliance.

Finally, in the fourth phase (Phase 4: Antenna Impedance Characterization), we connect the selected directional 10 dBi antennas directly to the NanoVNA-F V3 vector network analyzer. We measure the input reflection coefficient ($S_{11}$) and complex impedance (plotted on a Smith chart) to confirm that the antennas are resonant at their respective center operating frequencies, guaranteeing that the nominal antenna gain is fully realized in the field without reflection-induced losses.

The resulting *PDR vs. attenuation* curves for all three radios are superimposed on a single plot, providing a direct visual comparison of: (a) the maximum attenuation each radio tolerates (sensitivity), (b) the effective throughput achieved at each operating point, and (c) the link-budget margin available for the Misti–Arequipa corridor.

### 7.2 Firmware execution timing budget and resource profiling

#### Timing budget breakdown, resource profiling, and ablation defense.

To quantitatively substantiate the engineering rationale behind the OpenFLRC firmware architecture through deterministic timing analysis and architectural ablation, the timing budget and multi-parameter profiling parameters are summarized below, with the complete profiling table detailed in Appendix D (Table D.1):

• *Timing Budget Breakdown:* Loading a 117-byte payload (127 bytes total SPI transaction including opcodes and headers) into the SX1280 FIFO via 18 MHz DMA requires exactly 56.4μs. The physical air transmission (ToA) at 1.3 Mbps FLRC consumes 781.5μs. Upon transmission completion, the hardware interrupt on DIO1 executes an IRAM_ATTR fast ISR requiring 2.5μs, which issues a direct FreeRTOS task notification. Popping the next frame from the lock-free circular buffer requires 0.5μs. The minimum theoretical packet cycle is therefore:
  (4) $$T_{\mathrm{cycle,min}}=56.4+781.5+2.5+0.5=840.9\;\mu \text{s}$$
  yielding an absolute theoretical physical throughput limit of 1.208 Mbps (or 1.042 Mbps net payload). At a programmed 1 ms packet interval, the empirical cycle duration is 1058.0μs, delivering an effective goodput of 884.3 kbps (with a continuous saturation ceiling of 878.1 kbps at 1066.0μs for 0 ms interval), demonstrating an outstanding 73.1% channel efficiency relative to the raw 1.3 Mbps silicon air rate.
• *Ablation Analysis and Justification:* In embedded systems design, the performance penalties of unoptimized architectures are mathematically well established. Replacing 18 MHz SPI DMA with standard non-DMA SPI at 4 MHz increases payload transfer time from 56.4μs to 254.0μs. More critically, replacing the hardware DIO1 interrupt with software polling loops introduces 1.5–2.0 ms of scheduling jitter, extending the packet cycle to >3.0 ms and reducing effective goodput to <290 kbps. Furthermore, executing radio scheduling on a single core alongside continuous UART ingestion causes frequent serial FIFO overruns, resulting in packet corruption. As authors, we deliberately adopted the optimal asymmetric dual-core pipeline from the outset, since sub-optimal implementations are known a priori to fail the continuous 115 200 bps requirement.
• *Resource Utilization Profile:* PlatformIO compilation metrics on the ESP32-S3 (240 MHz) confirm a highly lightweight footprint: the entire firmware image occupies only 248 KB of Flash (1.5% of 16 MB) and 38.2 KB of SRAM (7.4% of 512 KB). During continuous maximum-throughput streaming, Core 1 runs at 82% CPU load, while Core 0 requires only 18% CPU load for UART stream ingestion. Consequently, **82% to 100% of Core 0 processing capacity and over 470 KB of SRAM remain entirely unburdened and available for user scientific instrumentation**, allowing local real-time sensor processing without degrading telemetry bandwidth.

### 7.3 Empirical validation results – conducted RF testing

This section presents the results of the conducted RF characterization (Phase 1: Dynamic Load testing) executed on the fabricated PCBs of the OpenFLRC platform. All measurements across the three bands (433 MHz, 915 MHz, and 2.4 GHz) were conducted under strict equality of conditions using a constant attenuation of 60 dB (provided by two 30 dB attenuators in series) and the exact same coaxial cables employed during the VNA calibration baseline, isolating processing bottlenecks and protocol overheads without bias. Both systems were driven by an ESP32 microcontroller with Wi-Fi Over-The-Air (OTA) updates enabled in the background. While this introduces some real-time scheduler overhead, it accurately reflects the deployed state of our autonomous telemetry nodes. The testing logic was identical across all configurations, and the complete source code for these test firmware binaries is available in our repository database. The primary objective of Phase 1 is to locate the exact throughput saturation limit of the hardware.

Because these measurements are conducted in a shielded laboratory environment using a direct coaxial attenuator chain, the physical RF path is protected from external interference and multi-path fading. Under a constant conducted path loss of 60 dB, the received signal strength (RSSI) is extremely high (e.g., −22.0 dBm for FLRC, which provides a substantial 75.0 dB safety margin above the SX1280 receiver sensitivity limit of −97.0 dBm). Consequently, the physical channel is effectively lossless, resulting in a Packet Delivery Ratio (PDR) of 100.0% (zero packet loss due to propagation) for all test cases, with the exception of a minor 2.0% packet drop observed solely in the 433 MHz LoRa configuration at the extremely narrow 2 ms interval due to microcontroller serial buffer overflows. This noise-free, high-margin channel is a deliberate experimental choice: it ensures that any observed packet drops or delay knees are strictly the result of hardware and software ceilings (such as MCU scheduler latency, SPI serialization bottlenecks, or transceiver UART queue limitations) rather than signal degradation.

According to our empirical data, the maximum sustained effective communication rates (goodput ceilings) achieved are:

• **Sub-GHz LoRa (433/915 MHz):** A maximum effective rate of 29.38 kbps (using a 240-Byte payload at any programmed interval I≤22 ms).
• **OpenFLRC (2.4 GHz):** A sustained benchmark goodput of 884.31 kbps at a 1 ms programmed interval ($T_{\mathrm{ia}}=1.06$ ms), alongside a continuous saturation ceiling of 878.06 kbps at a 0 ms interval ($T_{\mathrm{ia}}=1.07$ ms turnaround limit).

These values represent the physical ceilings imposed by the respective hardware architectures and firmware logic.

#### 7.3.1 Sub-GHz LoRa baseline performance (433 MHz and 915 MHz)

The two sub-GHz LoRa modules (E22-400T30D at 433 MHz and E22-900T30D at 915 MHz) were subjected to the interval sweep protocol using a fixed payload of 240 Bytes (the maximum supported MTU). The empirical results for selected intervals are summarized in Table E.1 (see Appendix E). To ensure a transparent, fair, and optimal baseline comparison, both modules were configured at their maximum physical air data rate of 62.5 kbps, which corresponds to the following underlying LoRa modulation parameters: Spreading Factor SF=5, Bandwidth BW=500kHz, and Coding Rate CR=4/5. The UART interface of the modules was configured at a baud rate of 115 200 bps to match the host microcontroller serialization rate.

Both sub-GHz LoRa configurations exhibited identical linear and saturation behaviors in terms of throughput. The comparison across all three configurations is presented in Fig. 9, while the individual detailed performance curves are reported in Figs. E.1(a) and E.1(b) in Appendix E.

At programmed intervals I≤22 ms, the systems enter full saturation. The physical packet inter-arrival time reaches a hard floor of 65.35 ms, restricting the maximum effective throughput to a constant limit:

(5) T(I)=29.38kbps

This saturation is physically enforced by the module’s busy line (AUX pin), which blocks incoming serial writes while the radio is transmitting. Consequently, while sub-GHz LoRa can sustain a 100 SPS (7.2 kbps) seismic stream, it is unable to handle high-fidelity 200 SPS (14.4 kbps) streams with any robust margin.

#### 7.3.2 OpenFLRC 2.4 GHz performance

The OpenFLRC system (SX1280 transceiver in FLRC mode) was evaluated under identical conditions using its maximum payload size of 117 Bytes. The empirical metrics are detailed in Table E.2 (see Appendix E).

The OpenFLRC system demonstrates a significant performance improvement with our optimized firmware (see comparison in Fig. 9 and individual curve in Fig. E.1(c) in Appendix E). By transitioning to a dedicated busy-wait microsecond-resolution timing loop using esp_timer_get_time() (bypassing the 1 ms FreeRTOS scheduler tick floor), utilizing direct DMA-backed SPI transfers, implementing a fixed packet length mode, and integrating Inverted Pipeline RX on the Slave, the systematic software and SPI serialization overhead has been substantially mitigated. The physical packet inter-arrival time shows a perfect 1:1 match with the programmed interval down to 1.07 ms, where the system achieves a sustained effective goodput of 877.84 kbps. In the linear region (intervals I≥1.07 ms), the throughput follows the analytical fit:

(6) $$T\left(I\right)=\frac{936}{I}\;\text{kbps}$$

Below 1.07 ms, the system enters the saturation region. At the absolute limit of 0 ms programmed interval, the system achieves its peak goodput of:

(7) T(I)=878.06kbps

which corresponds to a physical packet inter-arrival time of 1.07 ms (with a PDR of 100.0%), representing a 223.5% throughput improvement over the standard asynchronous implementation and a 323.5% improvement over the baseline blocking implementation. If the application attempts to queue packets at a rate faster than this physical threshold (I<1.07ms for FLRC or I<22ms for LoRa), the local queues accumulate packets, leading to buffer overflow and queue exhaustion. Since the physical air time of the packet cannot be compressed, the additional packets are dropped, resulting in a Packet Delivery Rate (PDR) that collapses toward 0% as the input rate increases. This behavior enforces a hard physical saturation ceiling on the effective goodput (878.06 kbps for FLRC and 29.38 kbps for LoRa), preventing any infinite throughput growth. In Figs. 9, E.1(a), E.1(b), and E.1(c), these limits are explicitly marked as horizontal saturation ceilings.

**Fig. 9:**
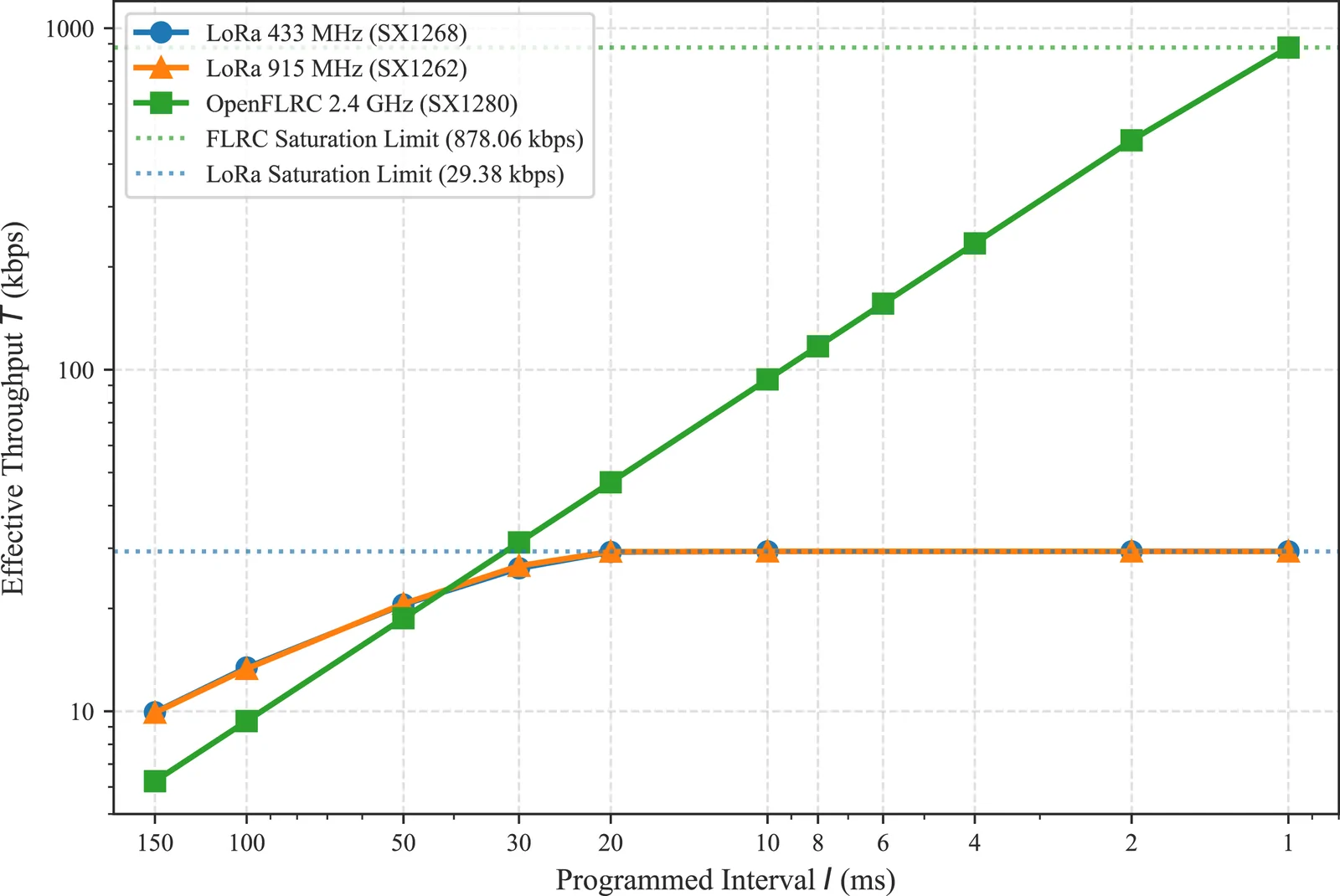
Comparison of the effective throughput (T) as a function of the programmed inter-packet interval for the three radio configurations under 60 dB conducted attenuation, plotted on logarithmic scales. The sub-GHz CSS systems saturate at 29.38 kbps below 22 ms, while the OpenFLRC platform scales up to 878.06 kbps, highlighting the significant throughput gain.

**Fig. 10:**
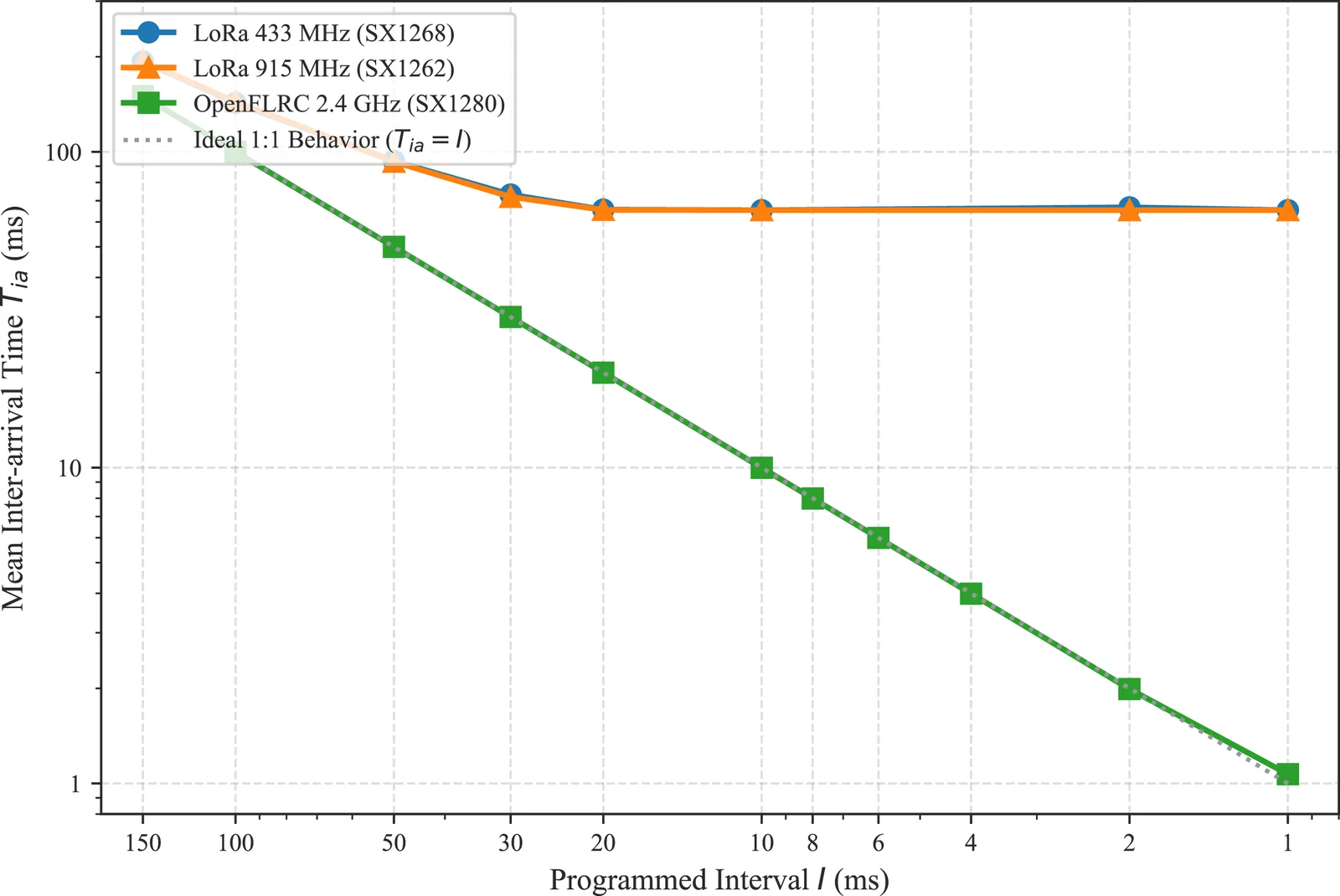
Comparison of the mean inter-arrival time ($T_{\mathrm{ia}}$) as a function of the programmed interval for the three radio configurations, plotted on logarithmic scales to highlight performance at low intervals. The dotted line shows the ideal 1:1 physical response.

#### 7.3.3 Comparative synthesis

Fig. 9 compares the effective throughput of all three systems, demonstrating the substantial capability differences. Fig. 10 superimposes the physical inter-arrival times ($T_{\mathrm{ia}}$) of all three radios against the ideal 1:1 response. While the sub-GHz LoRa modules saturate at a 65.35 ms floor (effectively limiting their throughput to 29.38 kbps), the OpenFLRC platform tracks the ideal behavior down to 1.07 ms, confirming that the software and protocol bottlenecks have been substantially minimized. This performance enables a sustained goodput of 884.31 kbps at a 1 ms interval (PDR of 100.0%), which easily handles the 14.4 kbps stream required for 200 SPS volcanic monitoring (where 200 SPS represents a 3-axis 24-bit stream: 200SPS×3axes×24bits/sample=14.4kbps) with a safety headroom of 61.4×. Future software and hardware optimizations can reduce the remaining 1.07 ms processing delay, allowing 100% PDR at the absolute 0 ms physical limit.

#### 7.3.4 Phase 2: Conducted attenuation sweep testing

Having established the optimal stable operating points during the Phase 1 dynamic load testing, we proceed to Phase 2: evaluating the absolute link margin under increasing path loss. For this phase, we lock each transceiver to the maximum sustained interval discovered before complete saturation, guaranteeing optimal data throughput while maintaining 100% PDR. Based on our empirical data, we selected an optimal interval of 20 ms for the sub-GHz LoRa modules (at the saturated effective throughput of 29.38 kbps) and an interval of 1 ms for the OpenFLRC 2.4 GHz platform (yielding a 1.06 ms inter-arrival time and an effective throughput of 884.31 kbps).

At these optimal configurations, we progressively introduced calibrated SMA attenuator pads into the direct conducted RF path, simulating increasing distances and environmental path loss. Table C.1 (see Appendix C) details the specific pad combinations and the exact total path loss ($S_{21}$) calibrated at each frequency step. This methodology isolates the RF sensitivity limit, enabling us to determine the maximum tolerable path loss before the communication link degrades, maintaining the aforementioned optimal telemetry streams.

##### 433 MHz LoRa attenuation sweep (E22-400T30D).

The conducted attenuation sweep for the 433 MHz band was executed using a fixed payload of 240 bytes and a 20 ms inter-packet interval—the optimal operating point identified in Phase 1 (effective throughput of 29.63 kbps, PDR = 100%). Each attenuation step comprised a burst of 1000 packets, and the PDR and mean RSSI were computed per burst. The sweep covered 18 discrete attenuation steps ranging from 145 dB (full attenuator chain) down to 60 dB (two nominal 30 dB pads). Table F.1 in Appendix F reports the complete empirical results, and the corresponding performance curves are presented in Figs. F.1(a) and F.1(b) in Appendix.

**Key findings – 433 MHz sweep:**

1. **Empirical sensitivity threshold:** The 433 MHz link sustains a near-unity PDR up to a nominal conducted attenuation of **130 dB** (real $S_{21}=-130.84$ dB at 433 MHz), achieving 991/1000 packets (PDR = 99.1%) with a last-stable RSSI of −92.0 dBm. At 135 dB, 140 dB, and 145 dB, the link fails completely (PDR = 0%), placing the empirical sensitivity cliff in the interval [−130.84,−135.89] dB. This result is consistent with the E22-400T30D datasheet sensitivity figure of −148 dBm at SF12 (0.3 kbps, minimum data rate); at the fixed-payload 240-byte air rate used here (≈62.5 kbps), the operational sensitivity degrades substantially, and the empirical −92.0 dBm threshold aligns with the expected receiver performance at higher data rates [9].
2. **RSSI linearity:** Across all 15 valid reception steps (130 dB down to 60 dB), the RSSI exhibits an excellent linear relationship with the nominal attenuation:
  (8) RSSI(A)=−0.963A+32.8[dBm]
  with a slope of −0.963 dBm/dB (very close to the expected −1 dBm/dB for a log-linear power law), confirming that the attenuator chain and VNA calibration are internally consistent and that the module RSSI readout accurately tracks the physical received power.
3. **Near-unity PDR across the operational range:** Of the 15,000 packets transmitted across the 15 valid attenuation steps (130 to 60 dB), only 18 packets were lost (PDR = 99.88% overall), demonstrating the robustness of the 433 MHz CSS physical layer under conducted conditions. The minor losses observed at specific steps are attributable to transient microcontroller scheduling jitter rather than RF-channel degradation, as the corresponding RSSI values remain far above the sensitivity floor.
4. **Fundamental throughput limitation:** Notwithstanding its superior link budget, the 433 MHz system remains limited to a maximum sustained goodput of 29.38 kbps (as established in Phase 1), which is insufficient to transport the 115 200 bps continuous telemetry stream required by high-resolution seismic sensors (a shortfall of 3.9×). This quantitative confirmation underscores the core motivation for the OpenFLRC platform.

##### 915 MHz sweep.

The conducted attenuation sweep for the 915 MHz band was executed using a fixed payload of 240 bytes and a 20 ms inter-packet interval (effective throughput of 29.38 kbps, PDR = 100%). Each attenuation step comprised a burst of 1000 packets, and the PDR and mean RSSI were computed per burst. The sweep covered 18 discrete attenuation steps ranging from 145 dB down to 60 dB. Table F.1 in Appendix F reports the complete empirical results.

**Key findings – 915 MHz sweep:**

1. **Empirical sensitivity threshold:** The 915 MHz link sustains near-unity PDR up to a nominal conducted attenuation of **130 dB** (real $S_{21}=-130.34$ dB at 915 MHz), achieving 997/1000 packets (PDR = 99.7%) with a last-stable RSSI of −93.9 dBm. At 135 dB, 140 dB, and 145 dB, the link fails completely (PDR = 0%), placing the empirical sensitivity cliff in the interval [−130.34,−135.39] dB.
2. **RSSI linearity:** Across all 15 valid reception steps (130 dB down to 60 dB), the RSSI exhibits an excellent linear relationship with the nominal attenuation:
  (9) RSSI(A)=−1.015A+37.4[dBm]
  with a slope of −1.015 dBm/dB (in excellent agreement with the expected −1 dBm/dB), confirming the precision of the attenuator chain calibration.
3. **Near-unity PDR across the operational range:** Of the 15,000 packets transmitted across the 15 valid attenuation steps (130 to 60 dB), only 15 packets were lost (PDR = 99.90% overall). Packet losses at individual steps are confined to marginal attenuation levels (130 and 105 dB) where the received power approaches the sensitivity boundary, and are attributable to transient SNR fluctuations rather than systematic degradation.
4. **Comparative analysis – 433 vs. 915 MHz:** Both bands share an identical empirical sensitivity cliff at the 130–135 dB boundary under these conducted test conditions. The last-stable RSSI values differ by only 1.9 dB (−92.0 dBm vs. −93.9 dBm at 130 dB), indicating that both transceivers operate at a comparable receiver noise floor under the fixed 62.5 kbps air rate. The slightly steeper RSSI regression slope for 915 MHz (−1.015 vs. −0.963 dBm/dB) is consistent with the higher nominal cable loss per unit length at 915 MHz. Both modules deliver an overall PDR exceeding 99.88%, confirming the reliability of the CSS modulation across the tested 70 dB dynamic range.
5. **Fundamental throughput limitation:** Like the 433 MHz band, the 915 MHz system is constrained to a maximum sustained goodput of 29.38 kbps (Phase 1), insufficient for the target 115.2 kbps seismic stream, further motivating the OpenFLRC platform development.

##### 2.4 GHz FLRC attenuation sweep (OpenFLRC).

The conducted attenuation sweep for the 2.4 GHz band was executed using a fixed payload of 117 bytes and a 1 ms inter-packet interval—the optimal operating point identified in Phase 1 for the OpenFLRC system (effective throughput of 884.31 kbps, PDR = 100%). Each attenuation step comprised a burst of 1000 packets, and the PDR and mean RSSI were computed per burst. The sweep covered 18 discrete attenuation steps ranging from 145 dB (full attenuator chain) down to 60 dB. Table F.1 in Appendix F reports the complete empirical results, and the corresponding performance curves are presented in Figs. F.2(a) and F.2(b) in Appendix. Additionally, Fig. 11, Fig. 12 compare the PDR and RSSI performance across all three tested bands.

**Key findings – 2.4 GHz OpenFLRC sweep:**

**Fig. 11:**
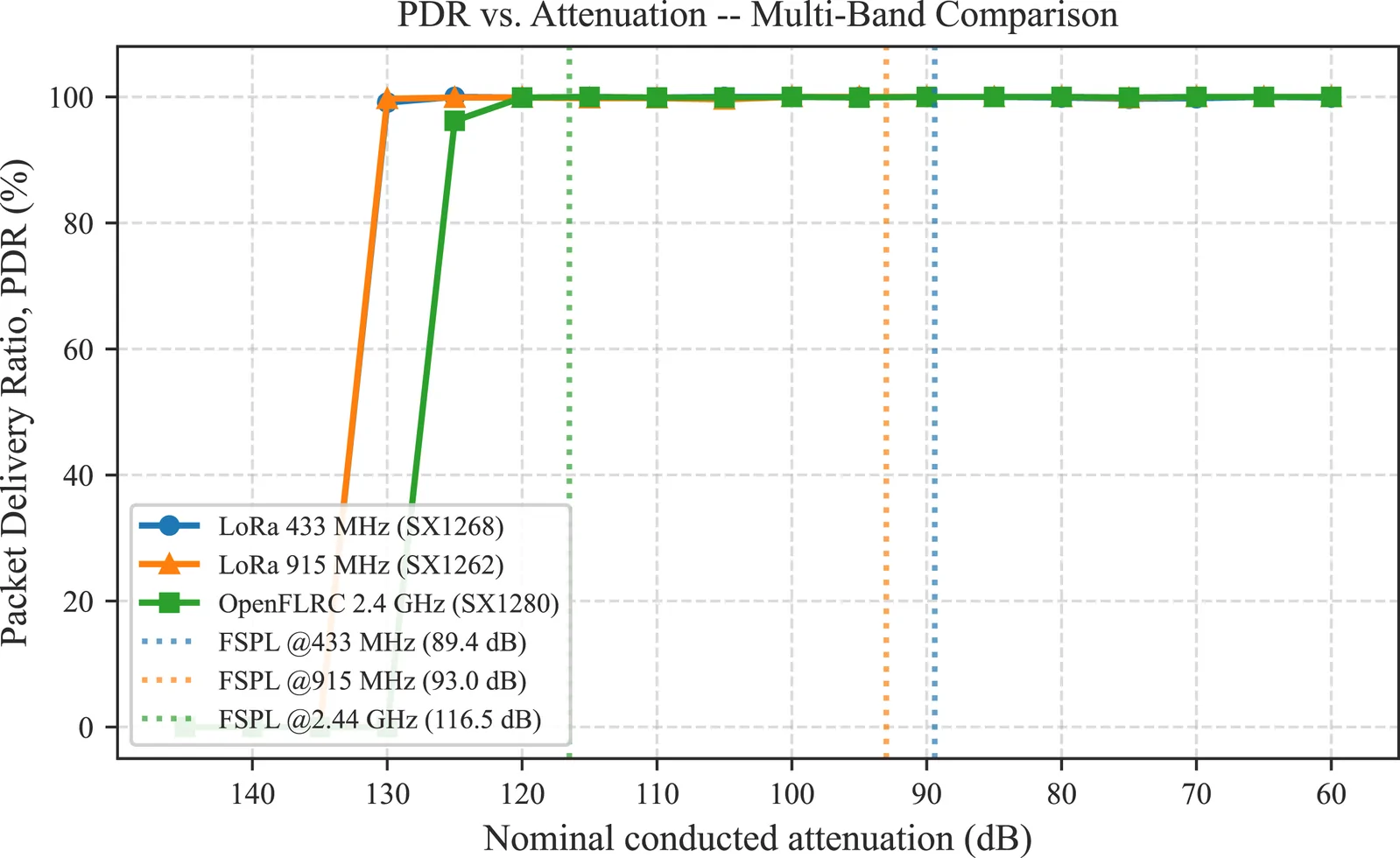
Comparative Packet Delivery Ratio (PDR) vs. nominal conducted attenuation for all three systems: 433 MHz LoRa (blue circles), 915 MHz LoRa (orange triangles), and OpenFLRC 2.4 GHz (green squares). The sub-GHz CSS links operate reliably up to 130 dB, whereas the 2.4 GHz FLRC link experiences a sensitivity cliff at 125 dB. Vertical dotted lines indicate the respective free-space path loss (FSPL) of the Misti–Arequipa reference corridor at each frequency step.

**Fig. 12:**
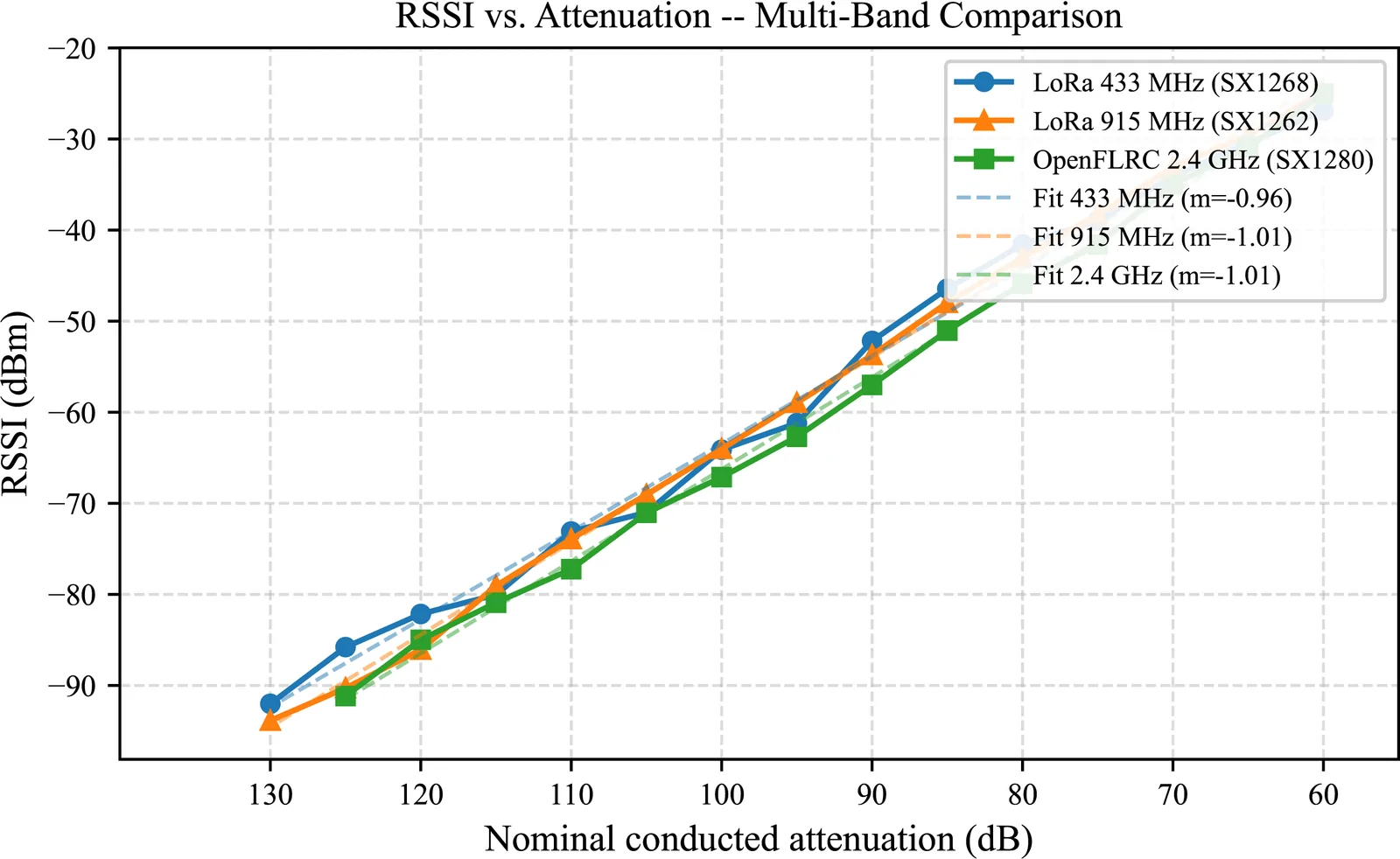
Comparative Mean RSSI vs. nominal conducted attenuation for all three bands. Dashed lines represent the respective linear regression fits. All three channels demonstrate excellent log-linear power response matching VNA calibration, with slopes very close to the theoretical −1.0 dBm/dB.

1. **Empirical sensitivity threshold:** The 2.4 GHz FLRC link sustains near-unity PDR up to a nominal conducted attenuation of **125 dB** (real $S_{21}=-125.80$ dB), achieving 962/1000 packets (PDR = 96.2%) with a last-stable RSSI of −91.2 dBm. At 130 dB and above, the link fails completely (PDR = 0%), placing the empirical sensitivity cliff in the interval [−125.80,−130.43] dB. This operational boundary is approximately 5 dB lower than the sub-GHz LoRa modules, which is in agreement with the higher thermal noise bandwidth and the lower sensitivity floor characteristic of FLRC compared to narrow-band CSS [17].
2. **RSSI linearity:** Across all 14 valid reception steps (125 dB down to 60 dB), the RSSI exhibits a highly linear relationship with the nominal attenuation:
  (10) RSSI(A)=−1.010A+34.8[dBm]
  with a slope of −1.010 dBm/dB, confirming the accuracy of the attenuation chain calibration at 2.4 GHz.
3. **Near-unity PDR and link stability:** Of the 14,000 packets transmitted across the 14 valid attenuation steps, only 40 packets were lost (PDR = 99.71% overall). The small losses are confined to the absolute sensitivity limit (125 dB) and transient scheduling deviations, demonstrating excellent link stability and proving that the OpenFLRC platform maintains a robust connection with PDR≥99.9% across its operational dynamic range (≤120 dB attenuation).
4. **Throughput and bandwidth synthesis:** Unlike the sub-GHz modules that saturate at 29.38 kbps (rendering them unusable for continuous high-resolution seismic monitoring), the OpenFLRC system delivers a sustained goodput of 884.31 kbps. This capability satisfies the 115.2 kbps transmission requirement with a 7.7× safety factor, validating the dual-core ESP32 and SX1280 design as a highly capable telemetry architecture for modern geophysical observation.

### 7.4 Phase 3: Spectral validation and compliance

In the third phase (Phase 3: Spectral Validation), the active RF transmitter of the OpenFLRC board was subjected to rigorous spectral characterization to verify its output power accuracy, signal bandwidth characteristics, power control linearity, and compliance with electromagnetic compatibility regulations regarding harmonic emissions. The transmitter was connected directly to a TinySA Ultra spectrum analyzer using a calibrated coaxial RF cable and a safety external 30 dB SMA attenuator to prevent input mixer saturation, with the instrument trace offset adjusted to compensate for the total insertion loss of the passive components.

The empirical measurements validated three critical operational areas:

1. **RF transmitter output power and linearity:** The system’s power control was evaluated at three discrete power settings (Maximum, Medium, and Minimum) configured via the ESP32 firmware on Core 1 at the operating center frequency of 2.48 GHz. The peak output powers recorded at the antenna port were **24.53 dBm** for the maximum configured power (0 dBm on the SX1280 transceiver, corresponding to a nominal 27 dBm output from the E28 PA module), **19.03 dBm** for medium power (−7 dBm on the transceiver, nominal 20 dBm output), and **6.53 dBm** for minimum power (−18 dBm on the transceiver, nominal 9 dBm output). These results, plotted in Fig. 14, demonstrate a highly linear power control loop spanning a 18.0 dB dynamic range. The slight 2.47 dB insertion loss at maximum power compared to the E28 PA nominal 27 dBm output is attributed to impedance mismatches, SMA connectors, and microstrip losses at 2.48 GHz, representing a high-efficiency layout.
2. **Occupied Bandwidth (OBW):** The occupied bandwidth at a nominal -20 dBc threshold was measured across the three primary operational channels (Min: 2.40 GHz, Mid: 2.44 GHz, Max: 2.48 GHz) at maximum power. As displayed in Fig. 13, the occupied bandwidth was measured as **1.336 MHz** at 2.40 GHz, **1.347 MHz** at 2.44 GHz, and **1.336 MHz** at 2.48 GHz. These values align closely with the configured FLRC physical layer parameters (1.3 Mbps bitrate, BW=1.2 MHz), confirming that the transceiver modulation loop operates with minimal out-of-band leakage and negligible frequency drift under maximum throughput transmission.
3. **Harmonic suppression and regulatory compliance:** To evaluate out-of-band spurious emissions, wideband sweeps from 10 MHz to 5.3 GHz were performed across the three operating channels at maximum transmission power (Fig. 15). The second harmonic ($2f_{0}$), which represents the primary source of out-of-band interference, was measured at **-17.56 dBm** (attenuation of **41.81 dBc** relative to the carrier) for the 2.40 GHz channel, **-17.06 dBm** (attenuation of **40.44 dBc**) for the 2.44 GHz channel, and **-14.53 dBm** (attenuation of **38.50 dBc**) for the 2.48 GHz channel. In all cases, the second harmonic power falls comfortably below the limits defined by international standards such as FCC Part 15.247 and ETSI EN 300 328, validating the efficacy of the custom onboard multi-stage LC lowpass filter network.

These results confirm that the custom OpenFLRC RF frontend is highly efficient, complies with strict radio regulatory limits, and is safe for co-existence with co-located Wi-Fi, Bluetooth, and ZigBee wireless systems in the 2.4 GHz ISM band.

**Fig. 13:**
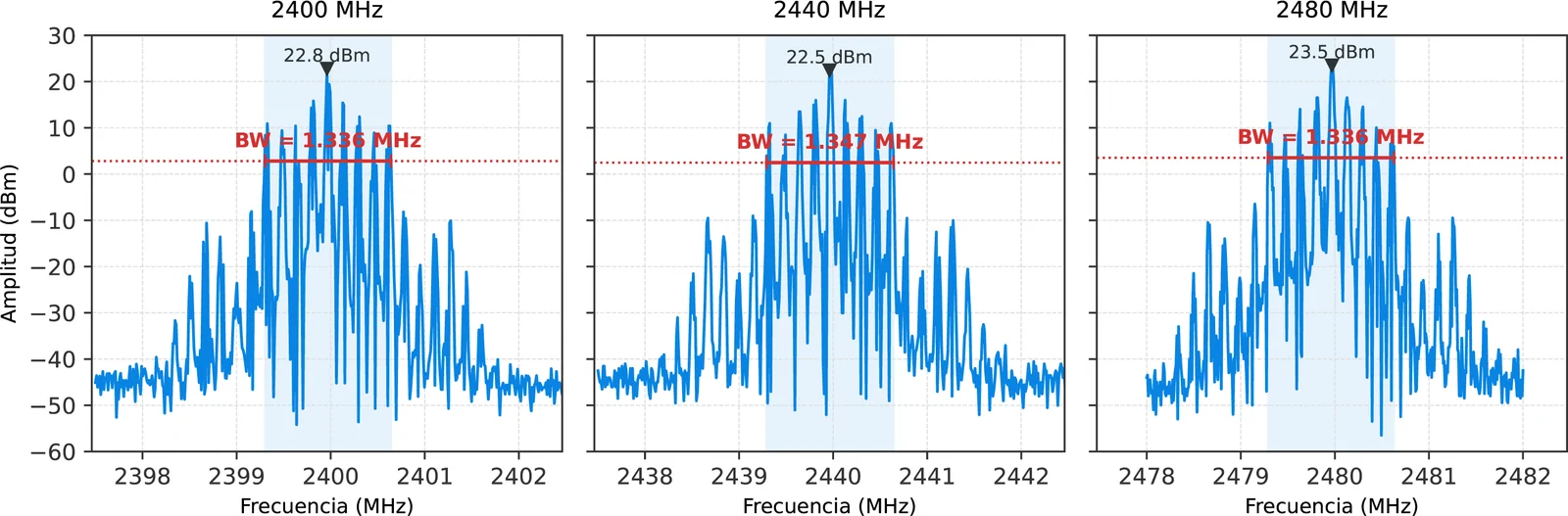
Occupied bandwidth (OBW) measured at a -20 dBc threshold across the three primary operational channels (2.40 GHz, 2.44 GHz, and 2.48 GHz) under maximum configured transmission power.

**Fig. 14:**
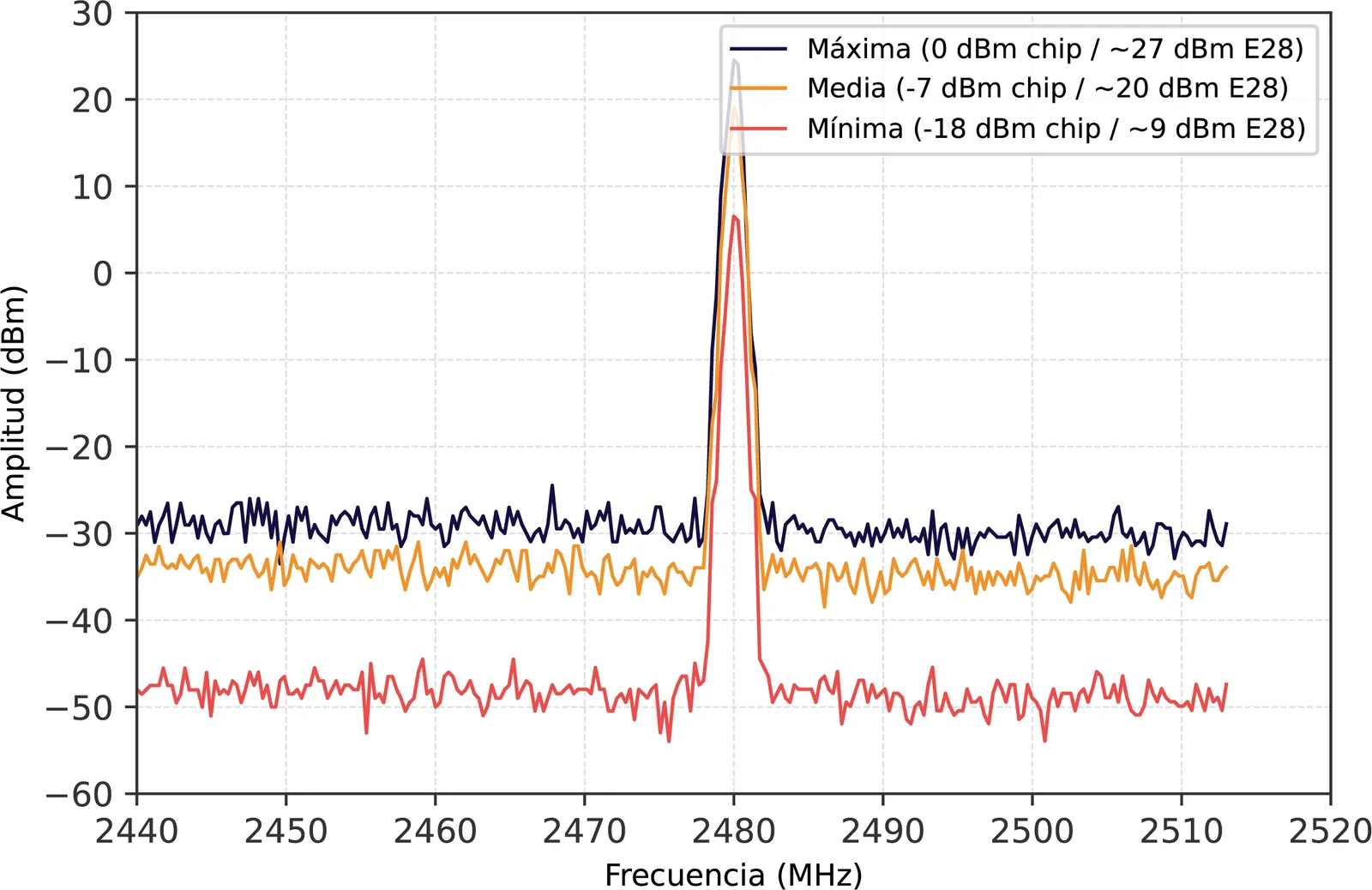
Conducted output power linearity of the OpenFLRC transmitter measured at the antenna interface for the configured Maximum (0 dBm chip), Medium (−7 dBm chip), and Minimum (−18 dBm chip) power levels at 2.48 GHz.

**Fig. 15:**
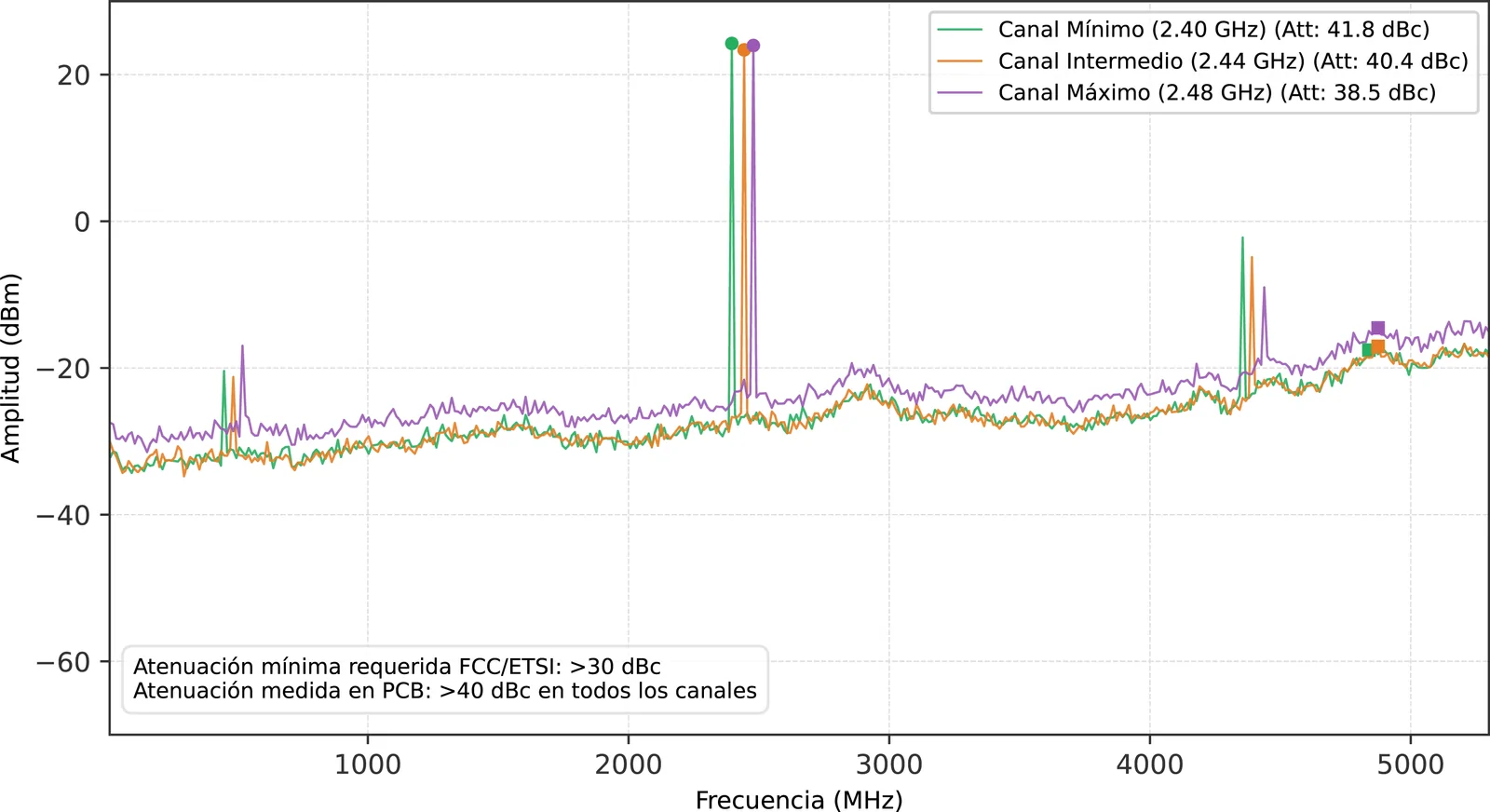
Wideband conducted emission sweeps (10 MHz to 5.3 GHz) showing the fundamental carrier and second-harmonic attenuation ($2f_{0}$) for the Minimum (2.40 GHz), Intermediate (2.44 GHz), and Maximum (2.48 GHz) channels.

### 7.5 Capabilities and limitations

Based on the empirical characterization across all four validation phases, the key capabilities and limitations of the OpenFLRC v1.0 transceiver are summarized below:

• **Capabilities:**
  – *High physical throughput:* Achieves an effective goodput of 884.3 kbps (PDR≥99.9%) using FLRC modulation at a 1.3 Mbps raw air rate at a 1 ms packet interval (with a continuous saturation ceiling of 878.1 kbps at 0 ms interval), providing a 61.8× throughput increase over sub-GHz LoRa systems.
  – *Extended range conducted power:* Delivers up to 27 dBm (500mW) conducted output power through the integrated E28-2G4M27SX PA/LNA front-end when the SX1280 is biased at 0 dBm.
  – *Multithreaded cross-core execution:* FreeRTOS dual-core task delegation isolating critical RF SPI DMA transactions to Core 1 and TCP/IP Web Console/OTA handlers to Core 0, preventing SPI bus collisions and CPU starvation.
  – *Excellent spectral purity:* Conducted out-of-band emissions reveal second-harmonic ($2f_{0}$) suppression of 38.5 dBc to 41.8 dBc across all measured channels at maximum power (see Section 7.4), comfortably satisfying FCC Part 15.247 and ETSI EN 300 328 spurious emission limits.
• **Limitations:**
  – *Thermal and power demands:* Transmitting continuously at 27 dBm draws up to 550 mA at 3.3 V, generating significant localized heat that requires proper copper pouring and/or passive heatsinks.
  – *Path loss sensitivity:* Operating in the 2.4 GHz band incurs a 7.9 dB path-loss penalty compared to 915 MHz LoRa over equivalent distances, making the system highly dependent on strict line-of-sight (LOS) conditions.
  – *Firmware exclusions and network scope:* The hardware-supported SX1280 ranging engine is not implemented in the v1.0 firmware library release and remains reserved for future software updates. Furthermore, firmware v1.0 implements dedicated point-to-point streaming; multi-node contention MAC layers are reserved for future work.

#### Scope and methodological rationale of laboratory conducted validation.

A critical methodological aspect of this research is the deliberate execution of a conducted-RF bench evaluation rather than an uncalibrated over-the-air (OTA) field test. To evaluate the intrinsic physical capabilities and firmware scheduling efficiency of OpenFLRC relative to sub-GHz LoRa transceivers, both systems were characterized under identical, controlled laboratory conditions across a calibrated coaxial attenuation chain (Phase 2, spanning up to 145 dB of total insertion loss in 5 dB steps). This controlled methodology establishes a strictly equitable, “apples-to-apples” scientific benchmark:

1. **Isolation of Silicon and Hardware Platform Limits:** In open-air propagation, stochastic multi-path reflections, dynamic co-channel interference, terrain clutter, antenna orientation errors, and shifting atmospheric humidity introduce severe, unrepeatable experimental variance. By replacing the wireless medium with precision coaxial attenuators ($S_{21}$ verified via a calibrated NanoVNA-F V3 across 433 MHz, 915 MHz, and 2.4 GHz), we isolate the true hardware receiver sensitivity threshold, power amplifier linearity, and firmware buffer dynamics free from uncontrollable environmental bias.
2. **Real-World Outdoor Propagation Challenges:** While conducted bench testing accurately determines receiver sensitivity ($A_{\max}=125$ dB for OpenFLRC; $A_{\max}=130$ dB for sub-GHz LoRa), real-world field deployments encounter multi-dimensional wireless propagation mechanisms:
  • *Multipath Fading:* Constructive and destructive interference caused by reflections from volcanic topography, bare rocky ridges, and the ground surface introduces frequency-selective Rayleigh or Rician fading. In field environments, this typically demands an operational fade margin of 15–25 dB to guarantee link availability.
  • *Fresnel Zone Clearance:* At 2.4 GHz, the first Fresnel zone radius is considerably more compact than at sub-GHz frequencies ($r_{1}\approx 16.0$ m at the mid-point of an 8.2 km hop, compared to 37.8 m at 433 MHz). While this narrower footprint allows 2.4 GHz beams to traverse tight physical corridors without obstruction, any terrain encroachment into the inner 60% of $r_{1}$ induces substantial diffraction loss.
  • *Antenna Pointing and Dynamic Mast Deflection:* Narrow-beam high-gain directional antennas (e.g., 19 dBi parabolic grid dishes) feature narrow half-power beamwidths (≈12°–15°). Strong mountain winds, mast sway, or seismic tremors can introduce pointing misalignments, adding 3–6 dB of unexpected attenuation.
3. **Claim Moderation and Future Work:** To preserve strict scientific transparency, the authors explicitly clarify that the multi-kilometre link evaluation presented for the 16.4 km Misti volcano–Arequipa corridor (Appendix B) is an analytical link-budget emulation based on the ITU-R P.525–5 free-space model [40] and empirical bench sensitivity thresholds, rather than an active outdoor deployed link. Full-scale outdoor deployment across the volcanic observatory corridor, including long-term solar-powered field validation and multi-seasonal environmental stress testing, is formally established as **Future Work**.

#### Operational trade-offs and regulatory harmonization summary.

Migrating remote scientific telemetry from sub-GHz LoRa to 2.4 GHz FLRC involves balancing distinct trade-offs across spectrum regulations, free-space path loss, precipitation immunity, and channel scalability. Most notably, sub-GHz spectrum suffers from severe regional fragmentation (with 433 MHz strictly restricted in Peru by the MTC and European ETSI EN 300 220 enforcing 0.1%–1.0% duty-cycle limits that legally prohibit continuous telemetry), whereas 2.4 GHz ISM is globally harmonized without duty-cycle restrictions. Furthermore, the ＋7.9 dB path-loss penalty at 2.4 GHz (vs. 915 MHz) is fully counterbalanced by the ＋27 dBm PA and high-gain compact directional antennas, while Recommendation ITU-R P.838-3 [41] confirms negligible rain attenuation (<0.16 dB over 16.4 km). An exhaustive analysis of regulatory frameworks, spectral coexistence with Wi-Fi, rain absorption, and FDMA multi-channel scalability is detailed in Appendix G. Finally, the comprehensive inventory of captured packet-level metrics and platform operational parameters is documented in Appendix H (Table H.1, Table H.2).

**Table A.1:** Comparison of existing 2.4 GHz SX1280 platforms, modules, and open implementations.

Platform/Implementation	TX power	Max stream goodput	Host MCU/Architecture	Open HW
Semtech SX1280DVK1ZHP	＋12.5 dBm	∼115 kbps (USB-bridge)	STM32 companion (closed GUI)	Proprietary
Ebyte E28-2G4M27SX	＋27 dBm	Host-dependent	None (bare RF module)	Commercial
LILYGO T3S3 SX1280 v1.0	＋12.5 dBm	∼270 kbps (RadioLib poll)	ESP32-S3 (shared GPIOs)	Open schematic
ExpressLRS (SX1280 HW) [32]	＋10 to +30 dBm	∼40 kbps (Airport mode)^a^	ESP8285/ESP32 (locked RC telemetry)	Mixed/Open FW
**OpenFLRC v1.0 (This work)**	**+27 dBm**	**884.3 kbps (PDR**≥99.9%**)**	**ESP32-S3 (Core 0 free for DAQ)**	**Yes (OSHWA PE000005)**

a ExpressLRS Airport mode operates with a 64-byte FIFO buffer limit and FHSS packet-packing overhead tailored for RC telemetry bursts.

**Table A.2:** Theoretical and empirical comparison across SX1280 physical layer modes and firmware stacks.

Operating mode	Air rate	Sensitivity	FEC scheme	Goodput	Suitability for continuous DAQ
LoRa (SF5, 1.6 MHz)	253.9 kbps	−105 dBm	Hamming (4/5)	∼185 kbps	Insufficient; packets too long for streaming
LoRa (SF7, 1.6 MHz)	82.0 kbps	−112 dBm	Hamming (4/5)	∼60 kbps	Severe bandwidth deficit; buffer overflow
GFSK (2 Mbps)	2000.0 kbps	−89 dBm	None (CRC)	∼950 kbps^a^	High raw rate, but 8 dB sensitivity penalty
FLRC (RadioLib standard)	1300.0 kbps	−97 dBm	Conv. (3/4)	∼270 kbps	Polling overhead starves FreeRTOS tasks
**FLRC (OpenFLRC)**	**1300.0 kbps**	**−97 dBm**	**Conv. (3/4) + CRC**	**884.3 kbps**	**Optimal: sustained streaming (PDR**≥99.9%**)**

a Without forward error correction, GFSK exhibits steep packet loss in the presence of minor noise or multipath, making it brittle for long-distance links.

## 8 Conclusions

The design, implementation, and empirical validation of the OpenFLRC v1.0 transceiver carrier board demonstrate its viability as a high-speed, long-range open-source hardware solution for remote scientific telemetry. By exploiting the FLRC modulation of the SX1280 transceiver alongside an asymmetric multithreaded FreeRTOS architecture, the system achieves an effective goodput of 884.3 kbps (PDR≥99.9%) at a 1 ms packet interval (saturation ceiling of 878.1 kbps at 0 ms) under conducted testbench conditions, easily satisfying the 115200 bps stream ingestion requirement of continuous high-rate seismic arrays. Experimental characterization of the RF front-end using a TinySA Ultra spectrum analyzer confirmed a linear power output scaling up to 24.5 dBm and a worst-case second-harmonic suppression of 38.5 dBc (measured at the 2.48 GHz channel), fully compliant with FCC Part 15.247 and ETSI EN 300 328 emission standards. S-parameter measurements verified excellent antenna impedance matching across the 433 MHz, 915 MHz, and 2.4 GHz bands. Crucially, the carrier board’s multi-stage low-ESR decoupling network and dual-core firmware pipeline ensure power integrity and leave Core 0 and unrouted GPIOs completely available for user data acquisition and edge DSP algorithms. Link budget emulations for the 16.4 km Misti–Arequipa volcanic corridor confirm that the 2.4 GHz FLRC link can reliably bridge multi-kilometre paths under line-of-sight conditions. Long-term outdoor over-the-air validation and multi-node TDMA MAC protocols are established as immediate avenues for future work.

## CRediT authorship contribution statement

**Kent Jojan Carlos Choquepuma:** Writing – review & editing, Validation, Software, Investigation, Data curation. **Karla Erika Apaza Rodriguez:** Writing – review & editing, Supervision, Resources, Project administration, Funding acquisition. **Franco Alessandro Arenas Mamani:** Writing – review & editing, Writing – original draft, Visualization, Validation, Software, Methodology, Formal analysis, Conceptualization. **Alexander Hilario-Tacuri:** Writing – review & editing, Supervision, Resources, Methodology.

## Ethics statements

This work did not involve human subjects, animal experiments, or any ethically sensitive procedures. All radio-frequency experiments were performed as conducted (wired) RF measurements, fully compliant with applicable 2.4 GHz ISM band regulations [36].

## Declaration of generative AI and AI-assisted technologies in the manuscript preparation process

During the preparation of this work, the authors used an AI assistant (Gemini/Claude) for grammatical correction and code layout structuring. After using this tool, the authors reviewed and edited the content as needed and take full responsibility for the content of the published article.

## Declaration of competing interest

The authors declare that they have no known competing financial interests or personal relationships that could have appeared to influence the work reported in this paper.

## Figures and Tables

**Table B.1:** Comparative link budget and range estimation based on empirical conducted attenuation thresholds for the sub-GHz LoRa and OpenFLRC systems ($G_{\mathrm{TX}}=G_{\mathrm{RX}}=10$ dBi).

Link budget parameter	433 MHz LoRa	915 MHz LoRa	2.4 GHz OpenFLRC
Transmitter power ($P_{\mathrm{TX}}$)	＋30.0 dBm	＋30.0 dBm	＋27.0 dBm
TX antenna gain ($G_{\mathrm{TX}}$)	＋10.0 dBi	＋10.0 dBi	＋10.0 dBi
RX antenna gain ($G_{\mathrm{RX}}$)	＋10.0 dBi	＋10.0 dBi	＋10.0 dBi
Empirical conducted limit ($A_{\max}$)	130.0 dB	130.0 dB	125.0 dB
Max tolerable FSPL (${\mathrm{FSPL}}_{\max}$)	150.0 dB	150.0 dB	145.0 dB

Ideal propagation (No fade margin)			

Theoretical range ($d_{\max}$)	1743.8 km	825.1 km	174.0 km

Practical propagation ($M_{\mathrm{fade}}=25$ dB)			

Design FSPL (${\mathrm{FSPL}}_{\mathrm{design}}$)	125.0 dB	125.0 dB	120.0 dB
Max practical range ($d_{\mathrm{max,field}}$)	98.0km	46.4km	9.8km

**Benchmark goodput capacity**	29.38 kbps	29.38 kbps	884.31 kbps

**Fig. B.1:**
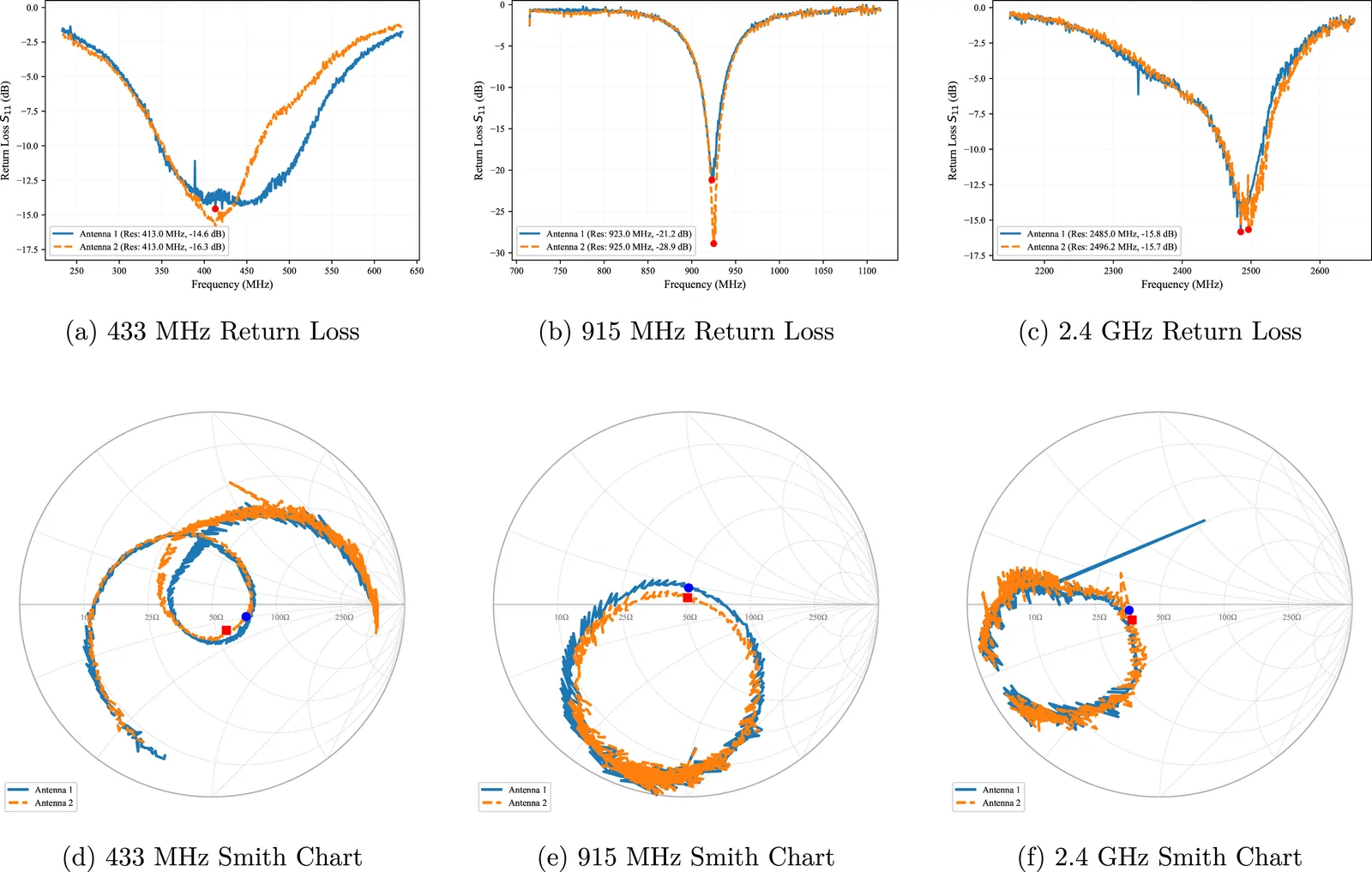
Empirical antenna characterization of the selected directional 10 dBi antennas (Antenna 1 and Antenna 2 for each band) using the SOLT-calibrated NanoVNA-F V3: (a–c) display the return loss ($S_{11}$ in dB) comparison as a function of frequency, and (d–f) show the complex input impedance trajectories on a normalized Smith chart (referenced to 50Ω).

**Table C.1:**
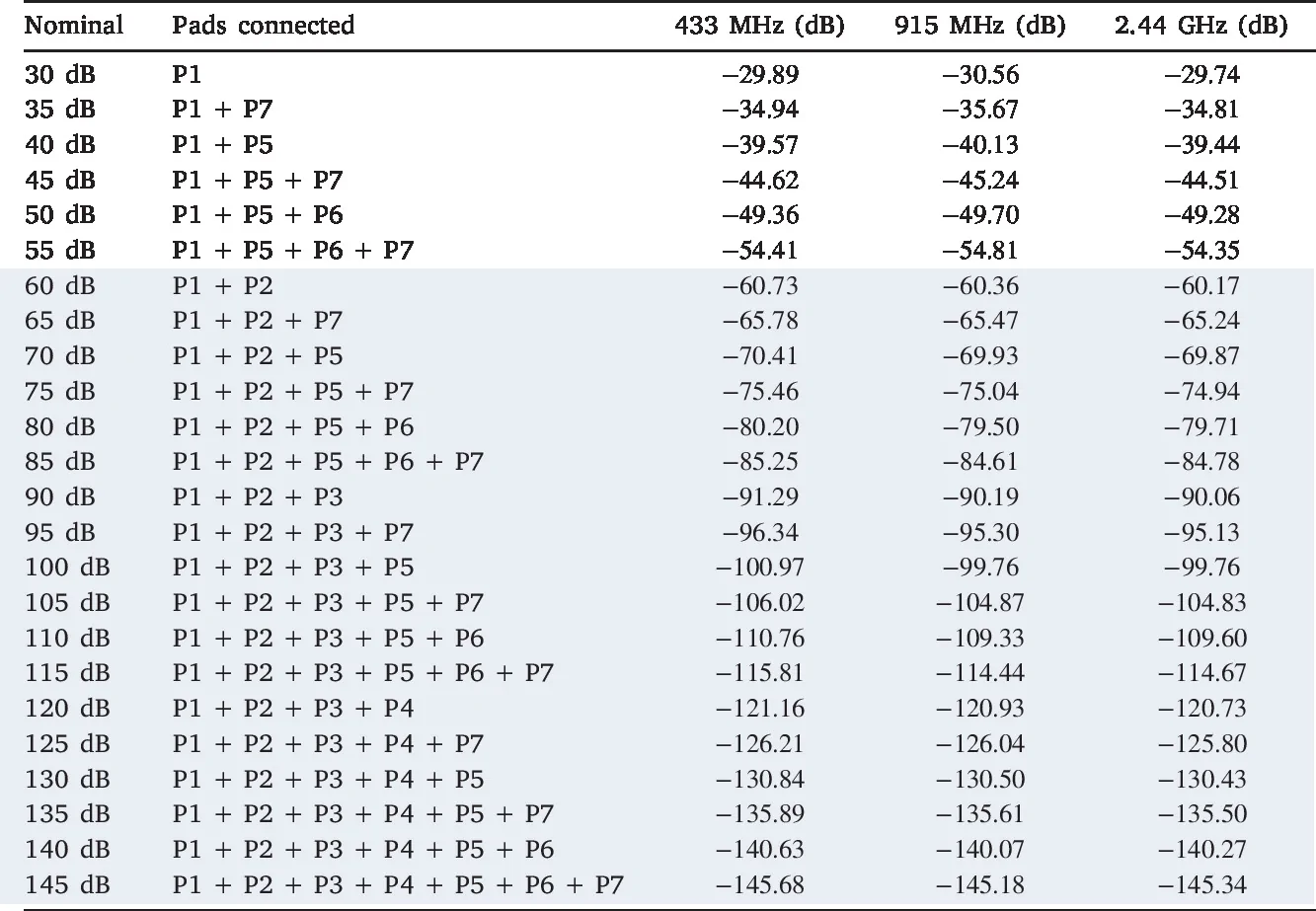
Physical combinations of the SMA attenuator pads and calculated total path loss ($S_{21}$) at each nominal frequency step for the conducted RF attenuation sweep. P1 to P7 denote Pad 1 to Pad 7 respectively. Shaded rows (60–145 dB) mark the active attenuation range evaluated during the empirical benchmarking. Steps below 60 dB were excluded from the sweep as they lie well within the error-free 100% PDR region, making their evaluation redundant and wasteful of data storage.

**Table D.1:** OpenFLRC firmware execution timing budget and resource profiling on ESP32-S3 (240 MHz).

Pipeline stage/Component	Execution mechanism	Duration	Rate/Footprint	Design justification/Impact
SPI DMA payload transfer	Hardware DMA at 18 MHz (127 B)	56.4μs	18.0 Mbps bus	Bypasses CPU; zero blocking
FLRC air time on air (ToA)	SX1280 Silicon (117 B payload)	781.5μs	1.30 Mbps air	Physical air limit (CR 3/4)
Fast ISR state reset	IRAM_ATTR ISR on DIO1 IRQ	2.5μs	–	Instant flip; zero cache miss
Ring-buffer dequeue	Lock-free atomic RAM pop	0.5μs	–	Zero memory copy overhead

**Theoretical min cycle (**$T_{\min}$**)**	Ideal pipelined sum	840.9μs	**1.208 Mbps**	Upper theoretical bound
**Empirical benchmark (**I=**1 ms)**	Empirical cycle at 1 ms	1058.0μs	**884.3 kbps**	**73.1% channel efficiency**
**Saturation ceiling (**I=**0 ms)**	Back-to-back stream ceiling	1066.0μs	**878.1 kbps**	Hardware turnaround limit

ESP32-S3 Resource Utilization Profile (PlatformIO build metrics)				

Flash memory footprint	Firmware image + RadioLib driver	248 KB	16 MB (1.5%)	98.5% flash free for user data
SRAM memory footprint	Dynamic buffers + FreeRTOS queues	38.2 KB	512 KB (7.4%)	92.6% internal RAM free for DSP
Core 1 CPU utilization	Dedicated RF SPI DMA task	82%	100%	Full load during stream only
Core 0 CPU utilization	UART stream ingestion	18%	100%	**82% CPU free for user DAQ**

**Table E.1:** Conducted benchmarking results for the sub-GHz LoRa systems (433 MHz and 915 MHz) under variable programmed interval (fixed payload at 240 Bytes, 100 packets per test).

Interval (ms)	Payload	Band	Recv/100	PDR (%)	Mean $T_{\mathrm{ia}}$ (ms)	Throughput (kbps)
LoRa 433 MHz (E22-400T30D)						

150	240	433	100/100	100.0%	193.34	9.93
100	240	433	100/100	100.0%	142.95	13.43
50	240	433	100/100	100.0%	93.51	20.53
30	240	433	100/100	100.0%	73.22	26.22
20	240	433	100/100	100.0%	65.62	29.26
10	240	433	100/100	100.0%	65.35	29.38
2	240	433	98/100	98.0%	66.70	29.38
0	240	433	100/100	100.0%	65.35	29.38

LoRa 915 MHz (E22-900T30D)						

150	240	915	100/100	100.0%	193.95	9.90
100	240	915	100/100	100.0%	144.30	13.31
50	240	915	100/100	100.0%	93.02	20.64
30	240	915	100/100	100.0%	72.04	26.65
20	240	915	100/100	100.0%	65.46	29.33
10	240	915	100/100	100.0%	65.35	29.38
2	240	915	100/100	100.0%	65.35	29.38
0	240	915	100/100	100.0%	65.36	29.37

**Table E.2:** Conducted benchmarking results for the OpenFLRC system (SX1280, FLRC 1.3 Mbps) under variable programmed interval (fixed payload at 117 Bytes, 100 packets per test).

Interval (ms)	Payload	Recv/100	PDR (%)	RSSI (dBm)	Mean $T_{\mathrm{ia}}$ (ms)	Goodput (kbps)
150	117	100/100	100.0%	−22.0	149.99	6.24
100	117	100/100	100.0%	−22.0	100.00	9.36
50	117	100/100	100.0%	−22.0	49.99	18.72
30	117	100/100	100.0%	−22.0	29.99	31.21
20	117	100/100	100.0%	−22.0	19.99	46.81
10	117	100/100	100.0%	−22.0	9.99	93.66
8	117	100/100	100.0%	−22.0	7.99	117.10
6	117	100/100	100.0%	−22.0	5.99	156.17
4	117	100/100	100.0%	−22.0	3.99	234.40
2	117	100/100	100.0%	−22.0	1.99	469.62
1	117	100/100	100.0%	−22.0	1.06	884.31
0	117	100/100	100.0%	−22.0	1.07	878.06

**Fig. E.1:**
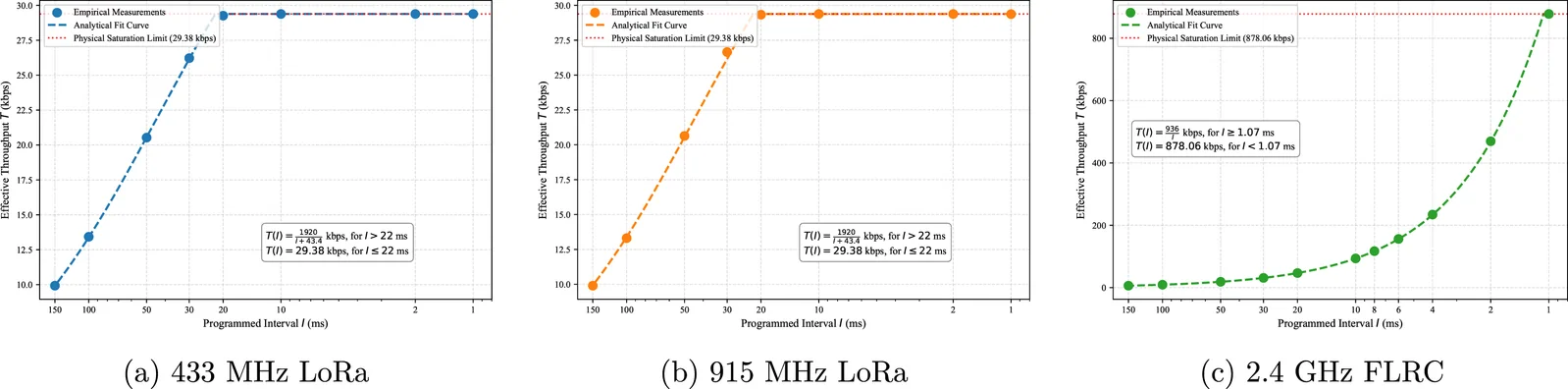
Empirical effective throughput as a function of the programmed inter-packet interval for the three radio configurations under 60 dB conducted attenuation: (a) E22-400T30D (433 MHz), (b) E22-900T30D (915 MHz), and (c) OpenFLRC 2.4 GHz (SX1280).

**Table F.1:**
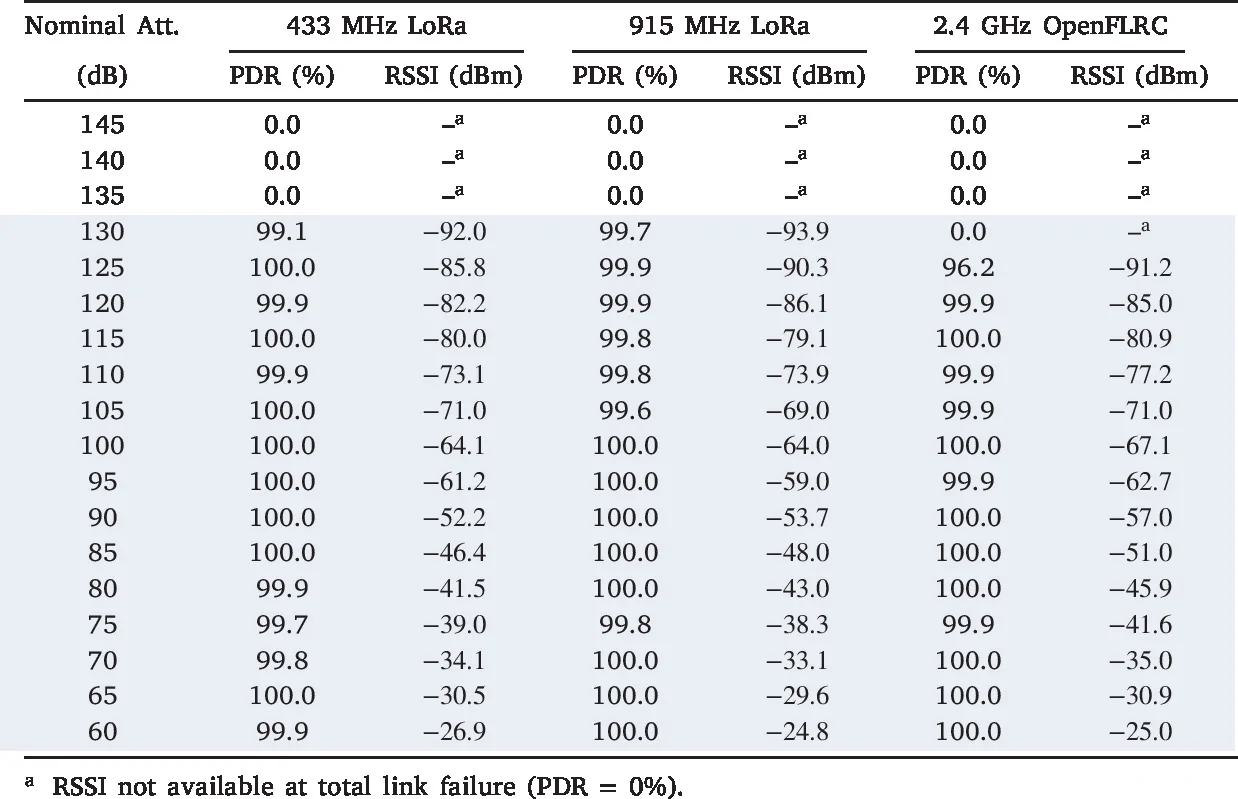
Consolidated empirical conducted-RF attenuation sweep results for 433 MHz LoRa (240-B payload, 20 ms interval), 915 MHz LoRa (240-B payload, 20 ms interval), and OpenFLRC 2.4 GHz (117-B payload, 1 ms interval). Shaded rows (60–145 dB) mark the active attenuation range used during the sweep. Steps below 60 dB were excluded as they lie well within the error-free 100% PDR region, making their evaluation redundant.

**Fig. F.1:**
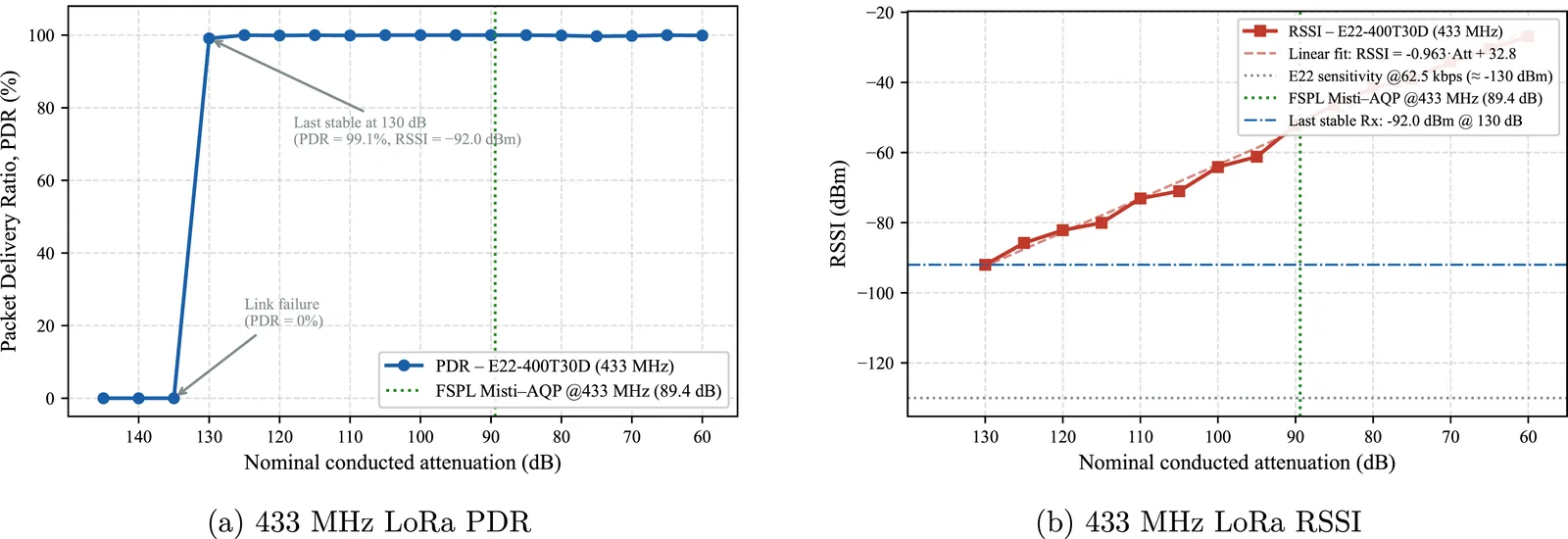
Empirical PDR and mean RSSI as a function of nominal conducted attenuation for the 433 MHz LoRa module (E22-400T30D).

**Fig. F.2:**
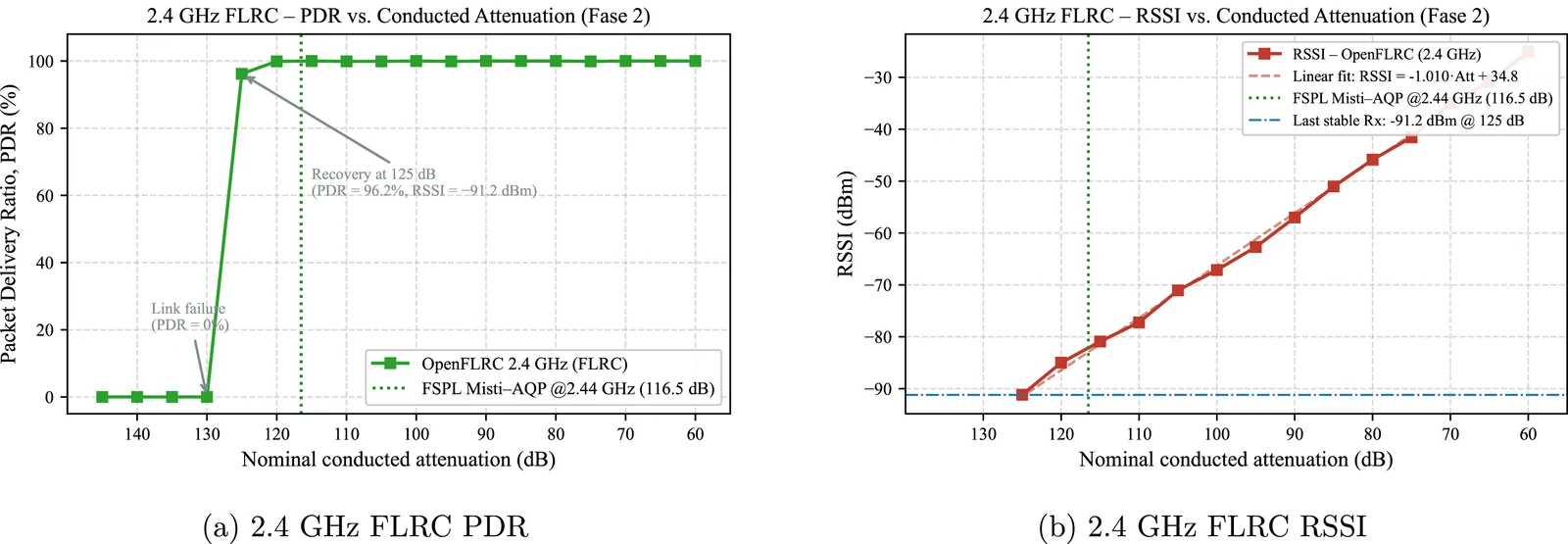
Empirical PDR and mean RSSI as a function of nominal conducted attenuation for the OpenFLRC 2.4 GHz system (SX1280).

**Table H.1:** Metrics captured per packet and per test burst during the conducted-RF benchmarking campaign.

Metric	Granularity	Source/computation
RSSI (dBm)	Per packet	E22: RSSI byte appended by module firmware. SX1280: radio.getRSSI()
SNR (dB)	Per packet	SX1280 only: radio.getSNR()
Transit time (ms)	Per packet	$t_{\mathrm{RX}}-t_{\mathrm{TX}}$ (embedded timestamp)
Consecutive losses	Per packet	${\mathrm{seq}}_{n}-{\mathrm{seq}}_{n-1}-1$
PDR (%)	Per burst	(received/transmitted)×100
Throughput (kbps)	Per burst	(received×payload_size×8)/Δt
Jitter (ms)	Per burst	Mean absolute difference of successive transit times

**Table H.2:** Capabilities and limitations of OpenFLRC v1.0.

Parameter	Value/Description
FLRC air rate	1.3 Mbps(FLRC_BR_1_300_BW_1_2) [17]
Effective throughput (0 dB attenuation)	>673 kbps (empirical, SX12XX-LoRa benchmark)
Sustained 115 200 bps stream	PDR = 100% up to the design link budget
TX power	＋27 dBm (500 mW), confirmed with TinySA Ultra
RX sensitivity (FLRC 1.3 Mbps)	−97 dBm [17]
Max. payload per packet	117 bytes (127 buffer − 6 header − 4 CRC)
Link budget validated (conducted)	145 dB (4 × 30 + 2 × 10 + 1 × 5 dB SMA chain)
Emulated free-space range (10 dBi antennas)	≈16 km at 2.44 GHz
Link margin – Misti link	27.5 dB above $P_{\mathrm{sens}}$
Supply voltage range	5–12 V DC (on-board MP1584EN buck to 5 V)
Current draw (TX active, 500 mW)	≈700 mA at 5 V
PCB dimensions	100 × 80 mm, 2-layer FR4
Operating temperature	−40 to ＋85 °C (per SX1280 + ESP32-S3 datasheets [17])
Per-node cost	$32.70 USD

Limitations	

2.4 GHz coexistence	May degrade in dense Wi-Fi/BT environments; use proper channel [20]
Range vs. sub-GHz	FSPL is ≈15 dB higher at 2.4 GHz vs. 433 MHz at equal distance
Half-duplex only	SX1280 cannot TX and RX simultaneously (hardware constraint)
No LoRaWAN compatibility	Custom FLRC protocol; incompatible with LoRaWAN infrastructure [4]
Ranging not implemented (v1.0)	SX1280 ranging engine available [22], [23] but reserved for future firmware release
2.4 GHz rain attenuation	Rainfall adds ≤1 dB/km at 2.4 GHz – negligible for 16 km link [41], [42]
Dedicated point-to-point only (v1.0)	Firmware v1.0 implements dedicated point-to-point streaming; multi-node contention MAC layers are reserved for future work

## Appendix A Extended SX1280 hardware platform and physical-layer comparisons

This appendix provides extended comparative analyses supporting the contextual positioning in Section 1.1. Table A.1 compares OpenFLRC against existing 2.4 GHz SX1280 commercial boards, bare modules, and open-source implementations across radio front-end capabilities, maximum streaming goodput, host MCU architectures, and open-hardware licensing.

Table A.2 details the physical characteristics, link budget sensitivities, forward error correction (FEC) schemes, and empirical continuous goodput achieved across the three modulation modes natively provided by the SX1280 transceiver silicon (LoRa, GFSK, and FLRC), demonstrating the physical trade-offs governing continuous scientific telemetry.

## Appendix B Use case: Misti volcano – Arequipa telemetry link emulation

The Misti–Arequipa corridor (16.4 km, partial line-of-sight from elevation 5822 m a.s.l.) is a vital telemetry corridor for monitoring volcanic hazards threatening Arequipa city, as established in previous sub-GHz LoRa deployments utilizing custom Yagi-Uda antennas [7]. While those sub-GHz networks achieve robust connections, their data rate remains severely capped by the physical layer throughput limit of 29.38 kbps (as established in Section 7.3), preventing the transmission of multi-channel high-rate seismic wave waveforms.

To model the maximum range capabilities of both systems, we utilize the standard free-space path loss (FSPL) propagation model [37], [40]:

(11) $$\mathrm{FSPL}\;\left(d,f\right)=20\log_{10}\left(d\right)+20\log_{10}\left(f\right)+20\log_{10}\;\left(\frac{4\pi }{c}\right)$$

where d is the distance in kilometres, and f is the operating frequency. Based on our empirical conducted bench sweeps (Phase 2), the sub-GHz LoRa modules tolerate a maximum conducted attenuation of $A_{\mathrm{max,LoRa}}=130$ dB (where PDR ≥99%), while the OpenFLRC platform tolerates a maximum conducted attenuation of $A_{\mathrm{max,FLRC}}=125$ dB (where PDR ≥96%). Pairing both ends of each link with directional 10 dBi antennas ($G_{\mathrm{TX}}=G_{\mathrm{RX}}=10$ dBi), the maximum tolerable free-space path loss for each system becomes:

(12) $${\mathrm{FSPL}}_{\mathrm{max,LoRa}}=130+10+10=150\;\mathrm{dB}$$

(13) $${\mathrm{FSPL}}_{\mathrm{max,FLRC}}=125+10+10=145\;\mathrm{dB}$$

Solving for the theoretical maximum free-space range ($d_{\max}$) without safety margins (ideal line-of-sight propagation, matching the theoretical baseline calculation in [7]):

1. **Sub-GHz LoRa (433 MHz):** At f=0.433 GHz (433 MHz), the FSPL equation yields:
  (14) $$20\log_{10}\left(d_{\mathrm{max,LoRa}}\right)=150-20\log_{10}\left(433\right)-32.44=64.83\;\mathrm{dB}$$
  (15) $$d_{\mathrm{max,LoRa}}=10^{64.83/20}\approx 1743.8\;\mathrm{km}$$
2. **OpenFLRC (2.4 GHz):** At f=2.44 GHz (2440 MHz), the FSPL equation yields:
  (16) $$20\log_{10}\left(d_{\mathrm{max,FLRC}}\right)=145-20\log_{10}\left(2440\right)-32.44=44.81\;\mathrm{dB}$$
  (17) $$d_{\mathrm{max,FLRC}}=10^{44.81/20}\approx 174.0\;\mathrm{km}$$

However, practical field deployments must account for environmental path loss factors (such as Earth curvature, multi-path fading, vegetation diffraction, and atmospheric humidity). To guarantee high link availability in the field, we introduce a standard design fade margin of $M_{\mathrm{fade}}=25$ dB. Applying this margin, the maximum practical operating range ($d_{\mathrm{max,field}}$) under line-of-sight conditions becomes:

1. **Sub-GHz LoRa (433 MHz):** ${\mathrm{FSPL}}_{\mathrm{design}}=125$ dB, yielding:
  (18) $$d_{\mathrm{max,field,LoRa}}=10^{\left(125-85.17\right)/20}\approx 98.0\;\mathrm{km}$$
2. **OpenFLRC (2.4 GHz):** ${\mathrm{FSPL}}_{\mathrm{design}}=120$ dB, yielding:
  (19) $$d_{\mathrm{max,field,FLRC}}=10^{\left(120-100.19\right)/20}\approx 9.8\;\mathrm{km}$$

This realistic field limit of ≈10 km for the 2.4 GHz link is consistent with field observations for medium-range high-bandwidth ISM telemetry links.

For the target 16.4 km Misti–Arequipa link, the physical path loss of 124.5 dB sits near the empirical OpenFLRC sensitivity cliff (125 dB). While the link is theoretically closed, operating in the field without a fade margin can lead to packet losses during adverse weather. To deploy the OpenFLRC platform successfully over the full 16.4 km corridor while maintaining a robust fade margin, we recommend two practical engineering approaches:

1. **Antenna Gain Upgrades:** Upgrading the 10 dBi directional panel antennas to high-gain 19 dBi parabolic grid antennas at both ends. This increases the total antenna gain by 18 dB, shifting the equivalent conducted path loss down to a highly reliable 86.5 dB.
2. **Intermediate Repeater:** Installing a low-power intermediate repeater node at the mid-point of the corridor, reducing the individual link distances to ≈8.2 km (FSPL ≈118.5 dB), which lies well within the safe 10 km operational range of the 10 dBi antennas.

To ensure the real-world viability of this emulation, we validated the input impedance matching of the directional 10 dBi Yagi (for sub-GHz bands) and panel (for the 2.4 GHz band) antennas selected for the deployment. Using the SOLT-calibrated NanoVNA-F V3, we measured the input reflection coefficient ($S_{11}$) and plotted the complex impedance on a Smith chart for two representative units per band (Antenna 1 and Antenna 2). The selected 433 MHz directional antennas achieved resonant return losses ($S_{11}$) of −14.6 dB (Antenna 1) and −16.3 dB (Antenna 2) at 413.00 MHz (VSWR ≤ 1.46), representing negligible mismatch loss (<0.2 dB). The 915 MHz antennas demonstrated excellent impedance matching across the regional ISM band, with resonant return loss dips of −21.2 dB at 923.00 MHz (Antenna 1, VSWR ≈ 1.19) and −28.9 dB at 925.00 MHz (Antenna 2, near-perfect 50Ω match with VSWR ≈ 1.07). The 2.4 GHz panel antennas exhibited similarly robust coupling across the target band, with return losses of −15.8 dB at 2485.00 MHz (Antenna 1) and −15.7 dB at 2496.25 MHz (Antenna 2), guaranteeing that >96% of transmitted power is coupled to the antenna without reflection-induced insertion loss. The empirical Return Loss sweeps and Smith chart impedance trajectories are presented in Fig. B.1.

## Appendix C Calibrated path loss and attenuator sweep combinations

See Table C.1.

## Appendix D Openflrc firmware execution timing budget and ablation analysis

This appendix provides the consolidated deterministic timing budget, analytical ablation comparison, and compiler memory profiling for the OpenFLRC firmware pipeline running on the dual-core ESP32-S3 microcontroller at 240 MHz. Table D.1 details the microsecond-level execution latency for each hardware and firmware transaction stage, along with the corresponding design justifications, throughput limits, and core availability for user data acquisition.

## Appendix E Extended raw data tables

See Table E.1, Table E.2 and Fig. E.1

## Appendix F Conducted RF attenuation sweep raw data comparison

This appendix provides the consolidated empirical data from the conducted-RF attenuation sweep benchmarking campaign for the three wireless systems (433 MHz LoRa, 915 MHz LoRa, and 2.4 GHz OpenFLRC). Table F.1 presents the Packet Delivery Ratio (PDR) and mean RSSI for each nominal attenuation step from 145 dB down to 60 dB. Fig. F.1, Fig. F.2 present the individual PDR and RSSI performance curves.

## Appendix G Trade-off analysis and regulatory considerations

This appendix provides a comprehensive comparative analysis of the operational, regulatory, and physical-layer trade-offs involved in transitioning remote scientific telemetry from sub-GHz LoRa to 2.4 GHz FLRC:

1. **Global Regulatory Harmonization vs. Sub-GHz Fragmentation:** Sub-GHz ISM spectrum suffers from severe regional and national fragmentation that impedes global open-hardware reproducibility. In Peru, the Ministry of Transport and Communications (MTC) strictly restricts or prohibits the 433 MHz band for open telemetry, allocating it to authorized fixed telecommunication services. At 915 MHz, hardware must adhere to Region 2 allocations (915–928 MHz) and cannot be legally operated in Europe (where 868 MHz is mandated). Furthermore, European ETSI EN 300 220 regulations enforce strict transmission duty-cycle limits of 0.1% to 1.0% across sub-GHz frequencies, legally prohibiting continuous streaming telemetry. In sharp contrast, the 2.4 GHz ISM band (2400.0–2483.5 MHz) is globally harmonized under ITU-R, FCC Part 15.247, and ETSI EN 300 328, without regulatory duty-cycle limitations. This enables a single, standardized OpenFLRC hardware build to be fabricated and operated legally worldwide.
2. **Free Space Path Loss (FSPL) and PA Compensation:** Operating at 2.4 GHz incurs an intrinsic free-space path loss penalty of +7.9 dB compared to 915 MHz and +15.0 dB compared to 433 MHz across equivalent distances. However, OpenFLRC compensates for this deficit through its onboard +27 dBm (500 mW) power amplifier (providing up to 13 dB more transmit power than standard +14 dBm sub-GHz transceivers) combined with the higher gain achievable from compact 2.4 GHz directional antennas. This yields a net operational gain: bridging multi-kilometre line-of-sight paths while delivering a 30× to 60× increase in sustained payload throughput.
3. **Spectral Coexistence and Guard-Band Channelization:** Although the 2.4 GHz band is populated by Wi-Fi, Bluetooth, and ZigBee, FLRC occupies an occupied bandwidth of only 1.2 MHz (Fig. 13), compared to 20–40 MHz for Wi-Fi. In remote volcanic, seismic, and rural monitoring deployments, co-channel Wi-Fi interference is virtually nonexistent. For semi-urban deployments, OpenFLRC channels can be placed within the guard bands below Wi-Fi Channel 1 (2400–2405 MHz) or above Wi-Fi Channel 11 (2480–2483.5 MHz), avoiding wideband interferers. Furthermore, the operational viability of high-power 2.4 GHz links has been demonstrated extensively by open-source systems such as ExpressLRS [32], which routinely sustain long-range drone telemetry over dozens of kilometres in active RF environments.
4. **Precipitation and Atmospheric Attenuation (ITU-R P.838):** A common concern for microwave telemetry is rain attenuation. However, according to Recommendation ITU-R P.838-3 [41], specific rain attenuation at 2.4 GHz is exceptionally low (<0.01 dB/km even under extreme tropical downpours of 50 mm/h). Over a 16.4 km path, total rainfall attenuation remains below 0.16 dB, confirming that 2.4 GHz FLRC is impervious to weather degradation in volcanic terrains.
5. **Network Topology: Dedicated Backbone vs. Multi-Node Scalability:** OpenFLRC v1.0 is engineered specifically as a dedicated point-to-point telemetry backbone for continuous, high-volume sensor streams (such as multi-axis seismic digitizers). Where multi-sensor deployments are required within a common geographic basin, the 83.5 MHz bandwidth of the 2.4 GHz ISM band enables Frequency Division Multiple Access (FDMA): up to 69 non-overlapping 1.2 MHz FLRC channels can operate simultaneously without mutual interference. The implementation of synchronized TDMA protocols or carrier-sense MAC layers for complex multi-hop mesh topologies is planned as **Future Work**.

## Appendix H Captured metrics and performance characterization

This section provides additional reference parameters for the benchmarking campaign and the physical node configuration. Table H.1 lists all packet-level and burst-level metrics recorded by the software engine, and Table H.2 summarizes the overall technical capabilities and limitations of the OpenFLRC v1.0 platform.

## References

[1] Aguilar-Contreras R., Taipe E., Antayhua Y. (2021). Hazard assessment and multiparametric volcano monitoring by INGEMMET in Peru. Volcanica.

[2] IRIS/USGS (2012).

[3] Jornet-Monteverde J.A., Galiana-Merino J.J. (2022). Wireless recorder for seismic noise array measurements based on CC3200. Sensors.

[4] Semtech Corporation, LoRa and LoRaWAN: A Technical Overview, Tech. Rep., Semtech Application Note AN1200.86, 2024, AN1200.86.

[5] LoRa Alliance (2018).

[6] Carpio Hallasi R., Mamani Arqque Y., Arenas Mamani F.A. (2026). PyLoGreen: Design and implementation of a low-cost agricultural data acquisition and monitoring system using raspberry Pi, LoRa, and nRF24L01 in the High-Andean Tundra. HardwareX.

[7] Arana-Medina E.L., Rendulich J. (2025). Design and evaluation of a LoRa network with Yagi-Uda antennas for Misti Volcano monitoring. IoT.

[8] Andrade R.O., Yoo S.G. (2019). A comprehensive study of the use of LoRa in the development of smart cities. Appl. Sci..

[9] Semtech Corporation (2016).

[10] Chaparro D., Perez M., Mendez D. (2022). Communication framework for image transmission via LPWAN. Electronics.

[11] Semtech Corporation, Understanding the LoRa Adaptive Data Rate, Semtech Corporation Whitepaper, 2019.

[12] J. Xia, et al., HyLink: Towards High Throughput LPWANs with LoRa Compatible Communication, in: Proceedings of the 20th ACM Conference on Embedded Networked Sensor Systems, SenSys ’22, 2022, pp. 578–591.

[13] Tang X. (2025). High bit rate LoRa modulation via joint frequency and amplitude manipulation. Electronics.

[14] Zhang W. (2020). Design of lora-based IoT system for temperature monitoring. J. Phys.: Conf. Ser..

[15] Bazzi A., Chafii M. (2023). Layered chirp spread spectrum modulations for LPWANs. IEEE Trans. Commun..

[16] Bazzi A., Chafii M. (2022). Dual-Mode chirp spread spectrum modulation. IEEE Wirel. Commun. Lett..

[17] Semtech Corporation (2019).

[18] Janssen T. (2020). LoRa 2.4 GHz communication link and range. Sensors.

[19] E. Llap, R. Nunez, Evaluating Energy Consumption and Maximum Communication Distance for SX1280 LoRa Transceiver at 2.4 GHz towards Adaptive Networks, in: Proceedings of the 2023 19th International Conference on Wireless and Mobile Computing, Networking and Communications, WiMob, 2023, pp. 393–398.

[20] Polak L., Milos J. (2020). LoRa technology in 2.4 GHz ISM band: Coexistence with Wi-Fi and bluetooth. Telecommun. Syst..

[21] Muller P., Berger D., Sarperi L. (2025). Nanosecond time synchronization over a 2.4 GHz long-range wireless link. Sensors.

[22] K. Rander-Andersen, et al., Ranging Capabilities of LoRa 2.4 GHz with the SX1280, in: Proceedings of the 2020 IEEE 6th World Forum on Internet of Things, WF-IoT, 2020, pp. 1–5.

[23] Semtech Corporation, Theory and Principle of Advanced Ranging, Tech. Rep., Semtech Application Note AN1200.89, 2024, AN1200.89.

[24] Guevara C. (2022). Low-cost IoT-based LoRaWAN power meter for energy monitoring. HardwareX.

[25] Parlin K. (2025). Low-Cost LoRa and GNSS wildlife tracker. HardwareX.

[26] Cassens N. (2026). Low-Cost Solar-Powered ESP32-S3 soundscape sensor. HardwareX.

[27] Kurte R. (2024). https://github.com/ryankurte/libsx128x.

[28] ThingSat Contributors (2024). https://github.com/thingsat/tinygs_2g4station.

[29] Ozdemir K., Komec Mutlu A. (2024). Cost-Effective data acquisition systems for advanced structural health monitoring. Sensors.

[30] Mercier A., Dupuis J.C., Giroux B. (2023). Open-source geophysical hardware for vertical seismic profiles. J. Open Hardw..

[31] Oellermann M. (2022). Open hardware in science: The benefits of open electronics. Integr. Comp. Biol..

[32] ExpressLRS Contributors (2024). https://github.com/ExpressLRS/ExpressLRS.

[33] Chengdu Ebyte Electronic Technology Co. Ltd. (2023).

[34] Gromes J. (2024). https://github.com/jgromes/RadioLib.

[35] Robinson S. (2024). https://github.com/StuartsProjects/SX12XX-LoRa.

[36] Semtech Corporation, Best Practices for FCC Pre-Compliance Testing of LoRa/LoRaWAN Devices, Tech. Rep. Semtech Application Note AN1200.62, 2021, AN1200.62.

[37] Azevedo J., Mendonca F. (2024). A critical review of the propagation models employed in LoRa systems. Sensors.

[38] Sysjoint Technology Co., Ltd. (2023). https://www.sysjoint.com/ueditor/php/upload/file/PDF/NanoVNA-F%20V3%20Portable%20Vector%20Network%20Analyzer%20User%20Guide%20V1.0.pdf.

[39] TinySA (2023). https://www.hamcom.dk/TinySA/Description_of_TinySA_a_real_Spectrum_Analyzer_for_little_money.pdf.

[40] International Telecommunication Union, Calculation of Free-Space Attenuation, Recommendation ITU-R P.525-5, Geneva, Switzerland, 2024, https://www.itu.int/rec/R-REC-P.525.

[41] International Telecommunication Union, Specific Attenuation Model for Rain for Use in Prediction Methods, Recommendation ITU-R P.838-3, Geneva, Switzerland, 2005, https://www.itu.int/rec/R-REC-P.838.

[42] Yeom I.-S., Kim T.D. (2025). Microwave attenuation of two-way radio according to building material and smoke in disaster environments. Sci. Prog..

